# Rheological Behavior and Processing of High-Performance Engineering Polymers

**DOI:** 10.3390/polym18172160

**Published:** 2026-09-04

**Authors:** Mohammod Hafizur Rahman, Md Ehtesamul Haque, Ziad Shatnawi, Md Arifuzzaman, Muhammad Ali Martuza, Amir Al-Ahmed

**Affiliations:** 1 Chemical Engineering Department, College of Engineering, Imam Mohammad Ibn Saud Islamic University (IMSIU), Riyadh 11432, Saudi Arabia; 2 Department of Computer Science, College of Computer Sciences & Information Technology, King Faisal University, Al-Ahsa 31982, Saudi Arabia; 3 Department of Civil and Environmental Engineering, College of Engineering, King Faisal University, Al-Ahsa 31982, Saudi Arabia; 4 Department of Computer Engineering, College of Computer, Qassim University, Buraydah 51452, Saudi Arabia; 5 Interdisciplinary Research Center for Sustainable Energy Systems, King Fahd University of Petroleum & Minerals, Dhahran 31261, Saudi Arabia

**Keywords:** Polyether Ether Ketone, rheology, injection molding, finite element analysis, high-performance polymers, thermo-mechanical coupling, process optimization

## Abstract

Advanced engineering applications increasingly demand high-performance polymers with exceptional mechanical and thermal properties; however, predicting their processing behavior remains challenging due to complex rheological responses and the lack of integrated experimental–simulation frameworks. This study introduces a novel integrated experimental–computational methodology that combines comprehensive rheological characterization, multi-model fitting, injection molding simulation, and multiphysics finite element analysis (FEA) to investigate the processing capabilities of Polyether Ether Ketone (PEEK) for aircraft bearing applications. Unlike conventional approaches that treat rheological analysis, processing simulation, and structural assessment separately, our framework establishes a coupled material–process–performance relationship through: (i) systematic thermal and mechanical characterization, establishing PEEK’s high melting temperature (343 °C), degradation temperature (575 °C), and tensile strength (95 MPa); (ii) comparative rheological model fitting, demonstrating that the Carreau–Yasuda model accurately predicts non-linear flow behavior with R^2^ = 0.97, outperforming simpler Power Law and Cross models; (iii) CAD-based injection molding simulation, revealing homogeneous flow distribution and optimized pressure profiles; and (iv) thermo-mechanical FEA, coupling thermal expansion with structural stress analysis to evaluate bearing integrity under operational conditions. The key novelty lies in the seamless integration of experimental rheology with multiphysics simulation, validated through rigorous statistical analysis achieving low RMSE (0.6854 MPa for stress, 0.003220 mm for deformation) and high correlation coefficients (R^2^ = 0.97). The results confirm a uniform flow distribution, stable structural performance, and reliable thermo-mechanical response, establishing PEEK’s suitability for high-performance aerospace components. This work contributes a comprehensive, scalable, and transferable framework that bridges experimental analysis and advanced simulation, enabling the predictive optimization of polymer processing parameters and significantly enhancing manufacturing reliability for industrial applications. The findings demonstrate the applicability of the experimental–computational analysis to the investigated PEEK bearing configuration under the specified processing and simulation conditions. Its specific contribution is the application of comparative rheological model fitting and experimentally characterized PEEK properties to the selected bearing geometry and processing conditions.

## 1. Introduction

Recent developments in the field of rheological analysis and high-performance processing of polymers have led to the enhanced design and application of these materials in different engineering applications. PEEK-based nanocomposites have been developed for high-efficiency electromagnetic wave absorption over a wide temperature range, demonstrating their potential for advanced functional and high-performance engineering applications [1]. Polyaryletherketone (PAEK) materials have been developed and are now being used in additive manufacturing, with a higher thermal resistance and mechanical strength that are appropriate for advanced manufacturing techniques [2]. The concept of compatibilization is essential for improving the mechanical, morphological and rheological properties of polyethylene/polypropylene blends modified by maleic anhydride-grafted polyethylene in a number of studies [3]. In the same way, polyamide 6-containing cellulose nanocomposites are a material with improved mechanical properties and processability on an industrial scale for melt processing [4]. The rheological and dynamic mechanical properties of polymers in fused deposition modeling have been studied to gain insights into their printing quality and adhesion between layers [5]. For industrial uses, some rigid-chain polymers like aromatic polyamides are the subject of research, as they possess superior strength and thermal stability [6].

Moreover, PEEK exhibits excellent mechanical strength, adhesive performance, and biocompatibility, making it suitable for high-performance engineering and biomedical applications [7]. The influence of fused deposition modeling (FDM) 3D printing parameters on the mechanical properties and microstructure of Carbon Fiber (CF)/PEEK and Glass Fiber (GF)/PEEK composites was investigated, showing that processing conditions significantly affect the strength and structural performance of printed thermoplastic composites [8]. The study of Ultra-High-Molecular-Weight Polyethylene (UHMWPE) fiber formation through the gel-spinning method has yielded a greater understanding of polymer physics and processing mechanisms [9]. Recent studies have demonstrated that material extrusion-based 3D printing techniques can be modified to successfully manufacture functional gradient PEEK components with improved structural performance and process adaptability [10]. Furthermore, the grafting and long-chain branching of polyamide 6 have improved the crystallization, foaming and mechanical properties of the material [11]. The incorporation of ceramic fillers in polymer composites has now enhanced their thermal conductivity and rheological properties, in particular for thermal interface materials [12]. At the same time, sustainable and high-performance materials have become more interesting through the use of bio-based and functional additives. The use of materials derived from lignin as alternatives for plastics has increased interest in this material, and it has been identified as a potential material for the additive manufacturing industry for sustainable material development [13].

Although considerable advances have been made in the area of rheological modeling, polymer blending and high-performance composite processing, there are still some challenges with the current methods, including incomplete linkage between rheology and processing behavior, inability to accurately predict under industrial conditions and limited integration of simulation with experimental validation. Most studies concentrate on property enhancement and not on a material–process–performance relationship, thus reducing confidence in their reliability in actual manufacturing situations. The key research challenge is therefore not the individual use of rheology or simulation, but the quantitative transfer of experimentally identified flow behavior into process and structural predictions for a specific PEEK bearing geometry. This study focuses on establishing how the experimentally determined shear-thinning response and selected constitutive model influence filling, pressure, thermal and thermo-mechanical predictions within the same processing chain. The proposed framework addresses these gaps with a comprehensive characterization of the rheological properties, modeling at different temperatures, and CAD–FEA-based process simulation for the realistic prediction of polymer behavior. The scientific focus is therefore placed on the PEEK-specific relationship between measured material behavior, processing response and bearing-level performance rather than on re-establishing standard polymer characterization principles. This study is different from traditional approaches as it creates a coupled approach between material properties, flow behavior and process parameters in a single predictive approach. The distinction lies in the explicit traceability of experimentally determined PEEK rheological behavior in the subsequent processing and structural analyses rather than in the availability of integrated simulation software itself. The framework uses comparative experimental model fitting to identify the most representative constitutive response before evaluating its implications for filling, pressure, thermal and structural behavior. The novelty is the integration of advanced rheological models, injection molding simulation and structural analysis of PEEK parts. The novelty is not attributed to the individual use of rheology, injection molding simulation, CAD or FEA, which are established practices in polymer engineering, but to their quantitative material-specific linkage within one PEEK bearing analysis. In particular, experimentally measured flow behavior is comparatively fitted using Power Law, Cross and Carreau–Yasuda models, and the selected constitutive description provides the material basis for subsequent process and thermo-mechanical predictions. More specifically, the contribution lies in quantitatively comparing constitutive descriptions and identifying the Carreau–Yasuda representation as the most suitable description of the measured non-linear PEEK flow response for subsequent processing analysis. The selected rheological representation is then carried through injection molding and thermo-mechanical simulations to connect flow characteristics with filling, pressure, stress and deformation behavior. This allows for an optimization of the processing parameters with mechanical and thermal reliability. The proposed approach offers a comprehensive and scalable high-performance polymer design approach for industrial applications.

### Organization of Paper

The rest of this paper is organized as follows:

✓ The related works in polymer rheology and high-performance engineering materials are presented in Section 2.
✓ The research gaps explored in this study are presented in Section 3.
✓ Section 4 describes the proposed methodology, including material selection, rheological modeling, processing simulation and FEA.
✓ The results and discussions are presented in Section 5, which includes the rheological behavior, thermal–mechanical properties, and results of simulations.
✓ Section 6 concludes the study and discusses future research directions for improving polymer processing and performance.

The article is structured into Section 1 (Introduction), Section 2 (Related Works), Section 3 (Problem Statement), Section 4 (Proposed Methodology), Section 5 (Experimental Setup), and Section 6 (Conclusions), with each section clearly presenting the background and research gap, related studies, problem formulation, proposed methodological workflow, experimental and simulation procedures, and concluding findings, respectively.

## 2. Related Works

Gul et al. [14] developed thermally conductive PEEK composites using twin-screw extrusion, rheological analysis, thermal characterization, and conductivity measurements; however, their study was limited by high filler loading complexity and a lack of scalability validation in diverse manufacturing conditions. Rodzén et al. [15] studied PEKK crystallization in Fused Filament Fabrication (FFF) using temperature control and kinetics analysis; however, their work was limited by a lack of scalability and parameter optimization. Sabet et al. [16] analyzed composite and nanocomposite polymers using advanced manufacturing processes like polymerization, additive manufacturing and mechanical/thermal characterization, but their study had some limitations in terms of scalability and lack of long-term testing. Jiang et al. [17] developed Polybutylene Adipate Terephthalate (PBAT)/Thermoplastic Starch (TPS) biodegradable composites using a two-step blending process with Epoxidized Soybean Oil (ESO) compatibilization, characterized by Fourier Transform Infrared Spectroscopy (FT-IR), Gel Permeation Chromatography (GPC), and mechanical testing; however, their study was limited by cost concerns and a lack of long-term biodegradation analysis.

Xiong et al. [18] developed mathematical prediction models using DSC and tensile analysis to study the crystallization and mechanical properties of PEEK/CF–PEEK; however, their work was limited by model accuracy constraints and a lack of complex processing condition validation. Maloney et al. [19] reviewed FFF processing of PEEK and PEKK focusing on process–structure–property relationships without a specific predictive model; however, their study was limited by a lack of experimental validation and insufficient analysis of PEKK processing behavior. Lucio et al. [20] studied polyurethane curing kinetics using rotational viscometry, dynamic rheometry, and kinetic modeling (first-order, autocatalytic, isoconversional), but practical industrial processing validation and scalability analysis were not performed. Lee et al. [21] developed a set of Conjugated Polymer (CP)-based ones with supramolecular/photochemical crosslinking and thermal–mechanical characterization, though their work still lacked large-scale recycling validation and long-term performance assessment.

Gavande et al. [22] manufactured a polymer blend of UHMWPE using melt blending and performed a rheological study and mechanical/thermal characterization; however, their work was limited in its discussion of mechanical/elongation properties and lacked large-scale processing validation. Li et al. [23] investigated the effect of hot compression molding parameters on PEEK using experimental analysis without a predictive model; however, their work was limited by a lack of advanced modeling and real-time process optimization. Herzog et al. [24] established analytical surrogate models to simulate melt conveying in single-screw extrusion, but the approximation assumptions made in the study and the resulting approximate accuracy limit the applicability under the very complex real industrial conditions. Baniasadi et al. [25] developed bio-based Polyamide–Functionalized Graphene Oxide (PA–FGO) nanocomposites using in situ polymerization, rheological testing, and mechanical/electrical characterization; however, the study was limited by a low filler concentration range and a lack of large-scale processing validation. Li et al. [26] have further demonstrated the mechanical performance of PEEK under advanced manufacturing conditions, supporting its suitability for demanding load-bearing applications and reinforcing the importance of processing–property relationships in PEEK components. Zheng et al.’s [27] studies on resin-based engineering composites have also demonstrated the importance of combining performance evaluation with predictive modeling for demanding mechanical and tribological applications. Xiang et al.’s [28] polymer composite processing studies have further shown that manufacturing conditions can strongly influence the resulting mechanical and structural properties, highlighting the importance of linking processing parameters with material performance. Li et al.’s [29] previous polymer failure studies have demonstrated that temperature, loading rate and stress state can significantly influence the predicted tensile response, emphasizing the importance of appropriate loading and thermal conditions in structural simulations. Duan et al. [30] showed that the tribological performance of polymer-based self-lubricating materials is strongly influenced by material architecture and lubrication mechanisms, highlighting the importance of considering friction and wear alongside mechanical integrity in bearing applications. Wen et al. [31] developed EMFF-2025, a neural network-based potential system for modeling energetic materials containing C, H, N, and O elements. The study demonstrated the applicability of machine learning for efficient and accurate material property prediction. Su et al. [32] investigated the combined effects of wettability gradients and pore structures in thermoresponsive polymer-functionalized fabrics. Their findings highlighted the potential of polymer-based structures for adaptive thermal and moisture regulation. Xie et al. [33] proposed a rapid aerodynamic analysis approach integrating the panel method with boundary-layer theory. The method enabled the efficient prediction of steady and unsteady aerodynamic characteristics. Pang et al. [34] applied machine learning to investigate pore-permeability evolution in fuel-related copolymers. The study demonstrated the usefulness of data-driven approaches for predicting complex material transport properties. Roy et al. [35] examined ballistic impact resistance in 3D-woven hybrid composites by considering damage confinement and layer architecture. The results emphasized the role of composite structural design in reducing backface deformation. Roy et al. [36] reconstructed ballistic impact temperatures in UHMWPE composites using morphology-calibrated thermal libraries. The approach provided an effective framework for estimating impact-induced thermal responses in composite materials. Cai et al. [37] developed a neural network model for predicting aerodynamic parameters of airfoils. The study demonstrated the effectiveness of neural networks in providing rapid aerodynamic predictions compared with conventional computational approaches.

The previous studies showed remarkable advances in polymer composites, rheological analysis, nanocomposites, biodegradable materials, and the simulation of processing using methods of extrusion, in situ polymerization, rheometry, thermal analysis, and numerical simulation. Most of the existing ones are, however, limited by their high processing complexity, limited optimization of the filler, limited industrial-scale validation, inadequate long-term performance analysis, and reduced accuracy of simplified models or experiment-only models under actual processing conditions. A further limitation is that experimental rheological observations are often not quantitatively traced through to component-level processing and structural responses within a single analysis chain. The present framework addresses this issue by using measured PEEK behavior and comparative constitutive fitting as the material basis for the subsequent flow and thermo-mechanical simulations of the bearing geometry. The proposed work will address these challenges by combining detailed rheological characterization, selection of optimized processing parameters and advanced simulation-based analysis using CAD and FEA software tools. The unresolved issue addressed here is therefore the quantitative consistency between experimentally obtained constitutive behavior and the predicted response of a specific PEEK component under processing and service-related conditions. Rather than treating rheological fitting, mold filling and structural analysis as separate outputs, the proposed sequence uses the experimentally supported flow representation as the basis for interpreting downstream process and performance responses. This hybrid method allows for precise material characterization, increases scalability and enables reliable performance evaluation in realistic injection molding conditions for high-performance engineering polymers.

## 3. Problem Statement

High-performance semicrystalline polymers showed a strong dependency on crystallization control during 3D printing, but lacked consistent control strategies across PEKK and PEEK grades, leading to unstable mechanical performance and limited process repeatability in additive manufacturing applications [15]. The prediction of crystallinity and mechanical behavior under non-isothermal molding was addressed, but existing models were limited by accuracy constraints under varying cooling rates and lacked robustness for complex reprocessing conditions in CF/PEEK systems [18]. Process–structure–property relationships in FFF-based PEKK and PEEK were explored, but insufficient quantitative modeling and limited processing window optimization restricted their applicability for reliable industrial-scale additive manufacturing [19]. The mechanical behavior of PEEK plates under different hot compression molding conditions was analyzed, but the study lacked predictive simulation integration and did not fully capture multi-parameter interactions affecting final material performance [23].

The suggested approach overcomes these challenges by combining rheological characterization, process simulation based on CAD models, and multiphysics analysis performed by FEA to provide a realistic prediction of the behavior of the material in actual processing conditions. This experimental–computational strategy helps to achieve a heightened degree of scalability, higher accuracy of the model, and more reliable performance prediction for high-performance engineering polymers in industrial applications. Simulation-derived filling, stress and deformation responses require experimental confirmation before their predictive capability can be established for practical manufacturing conditions. Therefore, the present analysis distinguishes experimentally measured material and rheological quantities from simulation-derived component responses and evaluates prediction reliability through quantitative error and correlation metrics.

### Objectives

- Investigate the rheological behavior and processing characteristics of PEEK for high-performance aircraft bearing applications to understand its flow and deformation behavior under industrial conditions.
- Utilize PEEK as the selected material for aircraft bearing systems, considering its superior thermal stability, mechanical strength, and suitability for high-load engineering applications.
- Evaluate the material through systematic material characterization, rheological testing, and rheological model fitting to determine its thermal properties, flow behavior, and predictive rheological parameters.
- Simulate the complete manufacturing process using injection molding simulation, CAD-based PEEK bearing modeling, and FEA to analyze flow behavior, structural integrity, and thermo-mechanical performance.

## 4. Proposed Methodology

**Figure 1:**
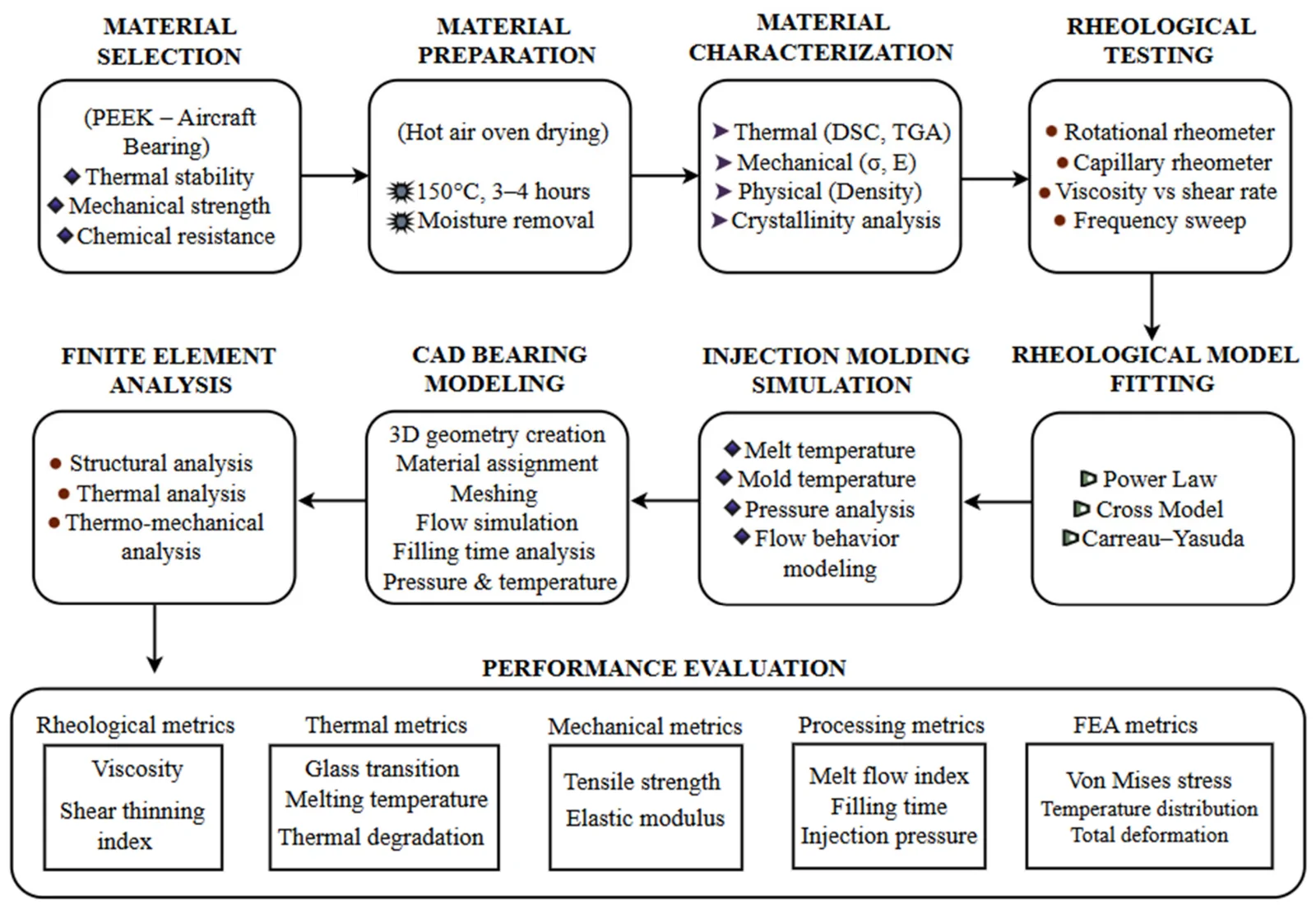
Proposed methodology framework.

The proposed methodology is a systematic approach to analyzing the rheological behavior and processing performance of high-performance engineering polymers, applied to the processing of aircraft bearing application with PEEK. In this study, the experimental investigation is specifically focused on Polyether Ether Ketone (PEEK) as the selected material for aircraft bearing applications. Accordingly, the material characterization, rheological analysis, processing simulation and FEA results are interpreted specifically for PEEK rather than for high-performance polymers as a general class. PEEK is first chosen for its excellent thermal, mechanical and chemical properties for use in the aerospace industry, and is then processed through a controlled material preparation process that includes hot-air oven drying to eliminate moisture and avoid defects during molding. Then, material characterization is conducted to assess various thermal properties (Tg, Tm, stability), mechanical strength, density and crystallinity to ensure suitability for high-performance applications. Rheological testing then takes place via the use of rotational and capillary rheometers to study viscosity, shear stress, and viscoelastic properties under various conditions. The experimental results are also fitted to the Power Law, Cross and Carreau–Yasuda equations in order to accurately model the flow behavior. The fitted constitutive response is used as the material-level basis for subsequent injection molding analysis, thereby maintaining continuity between measured rheology and simulated processing behavior. Model selection is based on the agreement between experimental and predicted viscosity responses, with the best-performing representation subsequently considered for process-level interpretation. Injection molding simulation is performed to predict the melt flow, filling behavior, pressure and temperature distribution under given injection molding conditions. A detailed CAD model of the PEEK bearing is created and coupled with simulations to study flow patterns, filling time and defect formation. Finally, FEA is used to assess structural, thermal and thermo-mechanical performance, including stress distribution, deformation and temperature variation, to achieve a comprehensive understanding of the material behavior and to ensure the optimal design and processing conditions. Figure 1 provides a concise visual overview of the experimental-to-computational workflow, while the subsequent sections focus only on the parameters and results necessary to reproduce and interpret each stage. The detailed scientific emphasis is placed on experimentally measured rheology, comparative constitutive-model fitting, injection molding predictions and thermo-mechanical FEA rather than repeating the workflow description.

Table 1 provides the principal experimental parameters used to characterize the PEEK material, including the parallel-plate rheometer configuration, thermal analysis conditions, ASTM D638 tensile-test parameters and Carreau–Yasuda model fitting criterion. The rheological measurements were performed over 360–400 °C and a shear rate range of 1–3000 s^−1^, while the tensile properties were obtained from five specimens under the specified ASTM D638 conditions.

### 4.1. Material Selection

High-performance thermoplastics are chosen due to their excellent mechanical, thermal and chemical characteristics, which support difficult engineering tasks. Of these, PEEK is selected for its outstanding performance properties. PEEK is a semi-crystalline high-performance engineering polymer that has high thermal stability, good mechanical properties and resistance to chemical and wear degradation.

These are the key features that have led to the choice of PEEK:

- Good thermal stability at high temperature;
- Excellent mechanical strength and stiffness;
- Excellent wear and chemical resistance;
- Suitability for aerospace applications such as aircraft bearing components.

The properties make PEEK very attractive for use in aerospace application, being favored for aircraft bearings and other components. Additionally, its high-temperature resistance and load-bearing capacity guarantee its reliability and durability in industrial settings.

### 4.2. Material Preparation

**Figure 2:**
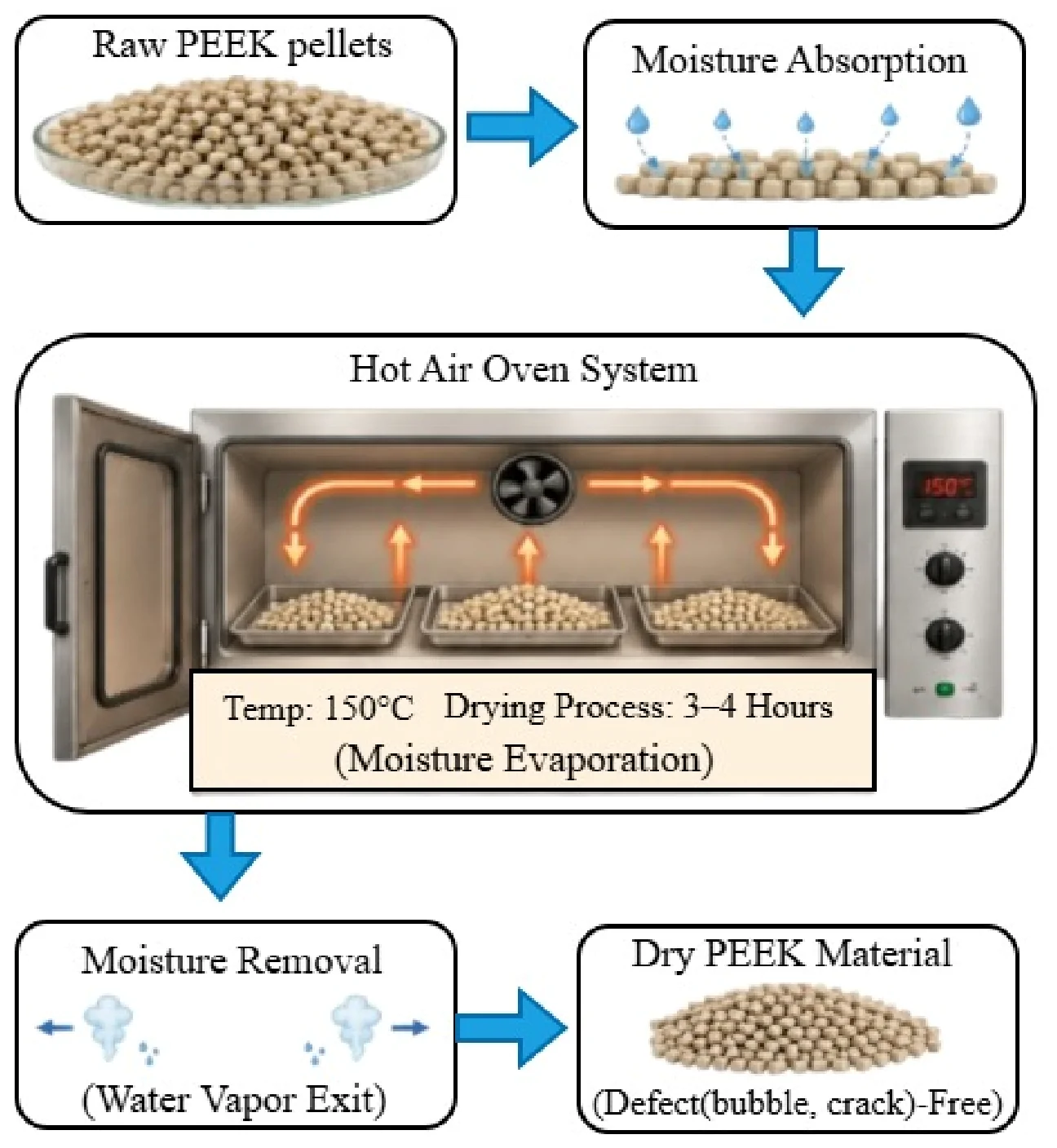
Material preparation process of PEEK using hot-air oven drying.

The pre-processing of polymers before manufacturing is known as material preparation. This primarily involves removing moisture and impurities to avoid defects in the production process which occur at high temperatures. Figure 2 shows the material preparation process of PEEK using a hot-air oven drying.

The first step is to gather the PEEK pellets in their raw state, and these can have moisture absorbed by the ambient air. This is eliminated by putting the pellets in a hot-air oven and drying them under controlled conditions for 4 h at 150 °C. In the oven, heated air is constantly circulated around the pellets to help distribute heat evenly and to help the moisture evaporate. The higher the temperature, the more the moisture absorbed by the PEEK pellets is removed from the product. This process guarantees the complete drying of the polymer, giving defect-free material for further processing. Water removal is important to prevent defects like voids, bubbles and thermal degradation in the subsequent injection molding process, improving the overall material stability and product quality. For the investigated PEEK bearing application, moisture control is particularly important because processing occurs at elevated temperatures and moisture-related defects can directly affect the reliability of the molded component. The reduction from 0.120% to 0.00099% moisture therefore establishes a controlled material condition before rheological and injection molding evaluation.

### 4.3. Material Characterization

Material characterization is the process of systematically identifying the thermal, mechanical and physical properties of a material to gain an understanding of its behavior under various conditions. This step ensures that the material selected is suitable in terms of performance parameters for processing and use. The fitted parameters showed narrow 95% confidence intervals, while the high R^2^ value of 0.97 and low RMSE and MAE values indicated good agreement between the rheological model and the experimental observations in Table 2.

The measured melting enthalpy of 72 J·g^−1^ corresponded to a crystallinity of 55.385%, indicating the calculated crystalline fraction used for subsequent material analysis in Table 3.

These material and thermo-mechanical parameters were used to define the PEEK material model and establish the loading and temperature conditions for the subsequent FEA in Table 4.

The thermo-mechanical response was evaluated by simultaneously considering the applied radial load and temperature-induced thermal effects using the defined material properties and boundary conditions in Table 5.

#### 4.3.1. Thermal Analysis

The thermal properties of the PEEK material are investigated using Differential Scanning Calorimetry (DSC) and Thermogravimetric Analysis (TGA). The glass transition temperature (Tg) and melting temperature (Tm) are used to determine the thermal transitions of the polymer and the used DSC method. TGA is employed to assess thermal stability by monitoring the material’s weight loss against temperature, which will indicate the degradation behavior at high temperatures.

#### 4.3.2. Mechanical Properties

Mechanical characterization is performed to evaluate the strength and stiffness of the material. The tensile strength is determined to find the maximum amount of stress that a material can resist before failing, and the elastic modulus is determined to understand the resistance of the material to the applied load.

#### 4.3.3. Physical Properties

Physical characterization includes determining the density of the material, which gives information about the compactness and mass distribution. Density is an important factor in the structural performance and efficiency of materials.

#### 4.3.4. Crystallinity Analysis

Crystallinity analysis is performed to assess the extent of ordered molecular structure in the polymer. The crystallinity will depend greatly on the degree of crystallinity, which is determined by applying the following Equation (1):

(1) $$X_{c}=\frac{\Delta H_{m}}{\Delta H_{m}^{0}}\times 100$$

In this equation, $X_{c}$ is the degree of crystallinity, $\Delta H_{m}$ is the measured melting enthalpy, and $\Delta H_{m}^{0}$ is the theoretical melting enthalpy. This analysis provides critical insight into the structure–property relationship of the material. For the PEEK bearing application, the measured Tg of 143 °C, Tm of 343 °C and Td of 575 °C define the thermal limits relevant to processing and subsequent service-oriented assessment. These experimentally determined temperatures provide the basis for selecting processing conditions while reducing the risk of interpreting flow and structural behavior outside the material’s stable thermal range.

### 4.4. Rheological Testing

This is conducted to measure the shearing and deformation characteristics of the molten PEEK material. The rheological measurements are carried out in a rotational and capillary rheometer that can be used to analyze the flow properties of polymer melts across the entire range of shear rates. PEEK material is heated to its melting point or above so that it will be fully molten before it is tested.

*Flow Behavior Analysis:* The primary rheological parameters measured include viscosity (η) and shear stress as a function of shear rate. These parameters provide insight into the resistance of the material to flow under applied forces. The variation of viscosity with shear rate is analyzed to determine the shear-thinning behavior of the polymer melt, which is a critical factor in processing performance. In the present PEEK system, this response is particularly relevant because viscosity reduction with increasing shear rate directly influences melt mobility during bearing cavity filling. The measured rheological behavior consequently provides the material-level evidence required to interpret the filling and pressure distributions obtained from injection molding simulation.

*Frequency Sweep Analysis:* Tests are performed to assess the material’s viscoelastic properties under oscillatory conditions. The analysis can be used to understand the material response in terms of storage modulus and loss modulus over frequencies, and can provide further information on molecular dynamics and structural stability during deformation.

### 4.5. Rheological Model Fitting

Rheological model fitting is a way of obtaining a mathematical model of the experimentally obtained flow behavior and enabling the description of the viscosity–shear rate relationship of the polymer melt. The three established constitutive models were considered only for comparative identification of the formulation that best represents the measured PEEK viscosity response over the investigated shear rate range. Their standard theoretical definitions are therefore not reproduced in detail, and the analysis focuses on model fitting accuracy and the resulting constitutive selection. By doing so, it is possible to predict the material response when subjected to various processing parameters. The models which are widely used are the Power Law model, the Cross model and the Carreau–Yasuda model. The purpose of comparing these established models is to determine which constitutive representation most closely captures the experimentally observed PEEK response under the investigated processing conditions. The comparison identified the Carreau–Yasuda model as the most accurate representation, achieving an R^2^ of 0.97 and providing the principal rheological basis for subsequent process interpretation.

1. *Power Law Model*

The Power Law model describes the non-Newtonian (shear-thinning or shear-thickening) flow behavior of polymer melts. It explains how the viscosity of a material changes with respect to the applied shear rate during flow. This law is defined as Equation (2):

(2) $$\eta =K{\dot{\gamma }}^{n-1}$$

Here, η denotes viscosity, $\dot{\gamma }$ denotes the shear rate, K denotes the consistency index, and n denotes the flow behavior index.

2. *Cross Model*

The Cross model is an advanced rheological model which is used to describe the viscosity curve of polymer melts in a wide range of shear rates, including low-shear and high-shear regions. It reflects a transition between the Newtonian region at low shear rates and the shear-thinning region at high shear rates. This model is represented by Equation (3):

(3) $$\eta =\frac{\eta_{0}}{1+(k\dot{\gamma }{)}^{m}}$$

where $\eta_{0}$ indicates zero-shear viscosity, $\left(k\dot{\gamma }\right)$ indicates a dimensionless term, and m indicates a dimensionless flow parameter.

3. *Carreau–Yasuda Model*

This model explains the complete flow behavior of polymer melts in the low-, intermediate- and high-shear regions. It is more accurate than simpler models because it models Newtonian and shear-thinning behavior. It is represented by Equation (4):

(4) $$\eta =\eta_{\infty }+\left(\eta_{0}-\eta_{\infty }\right)/{\left[1+(\lambda \dot{\gamma }{)}^{a}\right]}^{\frac{n-1}{a}}$$

In this equation, $\eta_{\infty }$ represents infinite-shear viscosity, $\lambda \dot{\gamma }$ represents a dimensionless parameter, a represents the Yasuda parameter, n denotes the flow behavior index, and $\frac{n-1}{a}$ controls the slope of the shear-thinning region.

### 4.6. Injection Molding Simulation

This is a numerical study of molten polymer behavior during a melting process inside a mold, used to predict the polymer processing responses and to optimize the processing parameters before polymer production. The choice of processing method for the PEEK material is injection molding, as it is suitable for high-performance parts. The main parameters of processing are determined: melt temperature, 360–400 °C, mold temperature, 160–200 °C; and injection pressure. All of these parameters have a significant impact on flow behavior, filling time and final part quality. To accurately describe the molten polymer flow during injection molding, the generalized momentum conservation Navier–Stokes equation for incompressible non-Newtonian flow is used, as shown in Equation (5):

(5) $$\rho \left(\frac{\partial v}{\partial t}+v\cdot \nabla v\right)=-\nabla p+\nabla \cdot \tau +F$$

Here, ρ is the density of the molten polymer, v is the velocity vector of flow, t is the time, *p* is the pressure inside the mold cavity, τ is the shear stress tensor, and F is the body force. The simulation analyzes melt flow progression, filling patterns, pressure distribution, and temperature variation during injection. For the selected aircraft bearing geometry, these outputs provide application-specific indicators of whether the PEEK melt can fill the cavity uniformly while avoiding localized pressure concentrations. The resulting flow-front and pressure patterns therefore translate the experimentally established material response into component-level processing information. These results help in optimizing processing conditions, preventing defects such as air traps and incomplete filling, and ensuring uniform mold filling with improved part quality and process efficiency.

### 4.7. PEEK Bearing CAD Modeling

**Figure 3:**
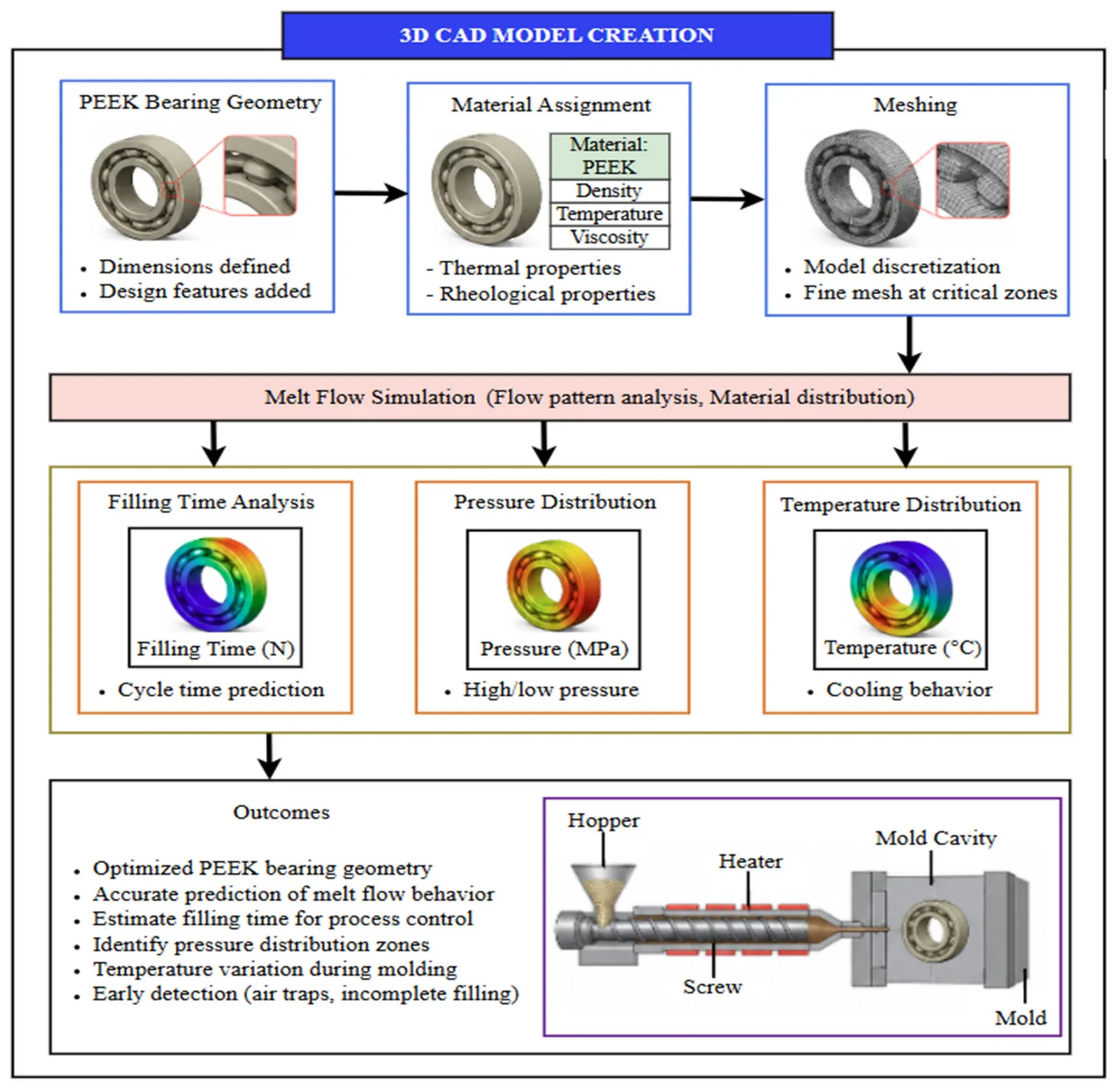
CAD-based injection molding simulation workflow.

This process includes the development of an accurate 3D geometric model of the bearing part in order to simulate and analyze the material flow and the behavior during injection molding. This step allows for the accurate visualization and prediction of the behavior of the molten polymer in the mold cavity. Figure 3 is retained as the CAD-to-injection molding workflow representation.

The first step in the PEEK bearing CAD modeling process is to develop a detailed three-dimensional geometric model of the aircraft bearing part using the proper CAD software, with all of the critical dimensions, shapes and design features correctly defined. Following this, the material properties of PEEK, such as its rheological and thermal properties, are assigned to the model to achieve realistic simulated behavior of the material and to accurately represent the flow of the molten polymer in the mold cavity. The developed model is then divided into finite elements using a meshing process, and a finer mesh is used in the areas that are important for the simulation to increase the accuracy of the simulation. This is followed by melt flow simulation to study the behavior of the molten PEEK flow through the mold to gain insight into the flow and distribution pattern of the material. From this simulation, the filling time needed to fill the entire cavity of the mold is calculated, an important step in optimizing production time. Furthermore, pressure distribution analysis is performed to understand the pressure distribution inside the mold, which can help in finding the high-pressure zone of the mold and possible defects. Lastly, the temperature distribution during the flow and cooling phases are studied to ensure uniform solidification and eliminate various thermal defects, increasing the quality and reliability of the final product.

### 4.8. FEA

FEA is a numerical simulation method for analyzing the structural and thermal properties of a component by breaking it up into smaller elements and solving the governing physical equations. In this study, FEA is specifically used to determine whether the predicted PEEK bearing response remains structurally and thermally acceptable under the defined loading conditions. The combined stress, deformation and temperature distributions identify the locations most susceptible to mechanical or thermal concentration within the bearing geometry. Based on the CAD geometry, a finite element model of the PEEK bearing component is built. The model is broken down into small elements, and suitable boundary conditions like loads, temperature and constraints are added to the model to mimic practical operating conditions.

1. *Structural Analysis*

The mechanical performance of the bearing is assessed using structural analysis when loads are applied. The stress distribution within the component is calculated using Equation (6):

(6) $$\sigma =\frac{F}{A}$$

Here, σ indicates stress, F indicates applied force, and A indicates cross-sectional area. The equivalent stress is often represented using the Von Mises criterion; this is defined in Equation (7):

(7) $$\sigma_{v}=\sqrt{\frac{{\left(\sigma_{1}-\sigma_{2}\right)}^{2}+{\left(\sigma_{2}-\sigma_{3}\right)}^{2}+{\left(\sigma_{3}-\sigma_{1}\right)}^{2}}{2}}$$

In this equation, $\sigma_{v}$ is the von Mises stress, and $\sigma_{1},\sigma_{2},\sigma_{3}$ are the principal stresses.

2. *Thermal Analysis*

This is done to check the temperature distribution in the component under operation. Fourier’s law is used to characterize the behavior of heat transfer, which is represented in Equation (8):

(8) q=−k∇T

where q is the heat flux vector, k is the thermal conductivity, and ∇T is the temperature gradient.

3. *Thermo-Mechanical Analysis*

This combines structural and thermal effects to analyze material behavior under multiple thermal and mechanical loadings. Thermal expansion is considered using Equation (9):

(9) σ=E(ϵ−αΔT)

Here, σ denotes stress, E denotes young’s modulus, ϵ denotes total strain, and αΔT denotes the thermal strain component. This is a comprehensive deformation, stress and failure analysis performed by combining the two analyses.

### 4.9. Computational Implementation and FEA Simulation Configuration

The proposed computational framework was implemented using MATLAB to perform data pre-processing, model development, parameter optimization, prediction, and finite element analysis. Table S1 summarizes the MATLAB functions, numerical settings, input–output operations, FEA configuration, and validation procedures used to ensure reproducible computational analysis. A detailed breakdown of the MATLAB functions, numerical settings, and FEA simulation configuration is provided in Table S1 of the Supplementary Materials.

## 5. Experimental Setup

This section establishes the organized sequence, equipment, parameters, and procedures used to run the simulations and conduct the experiments. This includes establishing testing conditions, choosing test equipment, establishing parameters, and conducting simulations or experiments to obtain reliable results. The moisture content decreased from 0.12 wt.% to 0.00099 wt.%, corresponding to a 99.175% reduction after the treatment in Table 6.

The repeatability of the processing conditions and numerical responses was evaluated using the mean, standard deviation, standard error, coefficient of variation, and 95% confidence interval shown in Table 7.

### 5.1. System Configuration

The hardware and software environment that is used to run simulations, modeling and analysis in the study is referred to as the system configuration. The detailed hardware and software specifications used for the realization of the computational and simulation tasks are presented in Table S2. The MATLAB toolboxes and functions used for simulation, model fitting, statistical validation and visualization are summarized in Table S3.

(A detailed breakdown of the hardware and software specifications is provided in Table S2 of the Supplementary Materials).

(A detailed breakdown of the MATLAB toolboxes and functions used in the analysis is provided in Table S3 of the Supplementary Materials).

### 5.2. Performance Evaluation Metrics

These metrics evaluate the overall performance and quality of the material and process through the evaluation of the results of the rheological, thermal, mechanical, processing and simulation tests.

#### 5.2.1. Rheological Metrics

Rheological metrics are used to characterize the flow characteristics of polymer melts by examining the relationship between the shear stress, shear rate, and viscosity during processing conditions.

➢ Viscosity (η) represents the resistance of a material to flow, that is, how easily the molten polymer flows during the injection molding process. η represents the viscosity, Aexp is the pre-exponential function, E is the activation energy for flow, and RT is the temperature effect.
➢ The shear-thinning index (n) represents the change in the viscosity of a material with shear rate and aids in the classification of the flow behavior of a polymer melt. It is expressed by Equation (10):

(10) $$n=\frac{dln\tau }{dln\dot{\gamma }}$$

Here, n indicates the flow behavior index, τ indicates shear stress, $\dot{\gamma }$ indicates the shear rate, and dln indicates the log scale.

#### 5.2.2. Thermal Metrics

These metrics are used to analyze the temperature-dependent behavior of polymers during transition, melting and degradation processes in relation to the thermal loading.

➢ The glass transition temperature (Tg) is the temperature at which a polymer transitions from a rigid glassy state to a flexible rubbery state, as measured by the change in heat capacity from DSC. This is denoted in Equation (11):
  (11) $$T_{g}=\frac{dC_{p}}{dQ}$$
  where $T_{g}$ denotes glass transition temperature, $dC_{p}$ denotes the heat capacity of the material, and dQ denotes heat flow supplied to the material.
➢ The melting temperature (Tm) is the temperature at which the crystalline part of the polymer is melted (as detected by the maximum heat flow during DSC). This is defined in Equation (12):
  (12) $$T_{m}=\mathrm{Temperature}\;\mathrm{at}\;\mathrm{peak}\;\left(\frac{dQ}{dt}\right)$$
  Here, $T_{m}$ indicates melting temperature, Q indicates heat flow, t indicates time, and $\frac{dQ}{dt}$ indicates the rate of heat flow.
➢ The thermal degradation temperature (Td) is the temperature at which the polymer begins to decompose, determined from the TGA peak of the maximum mass loss coefficient. It is represented by Equation (13):
  (13) $$T_{d}=\mathrm{Temperature}\;\mathrm{at}\;max\left(\frac{dm}{dt}\right)$$
  where $T_{d}$ is the thermal degradation temperature, m is the mass of the material, and $\frac{dm}{dt}$ is the rate of mass loss.

#### 5.2.3. Mechanical Metrics

These metrics are used to evaluate the strength and stiffness of materials by analyzing their response to applied forces and deformation.

➢ Tensile strength: A material’s strength is the maximum stress that it can endure (without failing) when it is being stressed. This is expressed in Equation (14):
  (14) $$\sigma_{u}=\frac{F_{max}}{A_{0}}$$
  Here, $\sigma_{u}$ indicates ultimate tensile strength, $F_{max}$ indicates maximum applied force before fracture, and $A_{0}$ indicates the original cross-sectional area.
➢ Elastic modulus: The elastic modulus is a measure of the stiffness of a material, which is resistance to elastic deformation when subjected to stress. This is expressed in Equation (15):
  (15) $$E=\frac{d\sigma }{d\epsilon }$$
  In this equation, E is the elastic modulus, and $\frac{d\sigma }{d\epsilon }$ is the slope of stress–strain curve.

#### 5.2.4. Processing Metrics

These are used to analyze the parameters which control mold filling, pressure and manufacturing efficiency during polymer manufacturing to evaluate flow behavior and manufacturing performance.

➢ The melt flow index (MFI) refers to the ease with which the molten polymer flows under normal conditions. This is represented in Equation (16):
  (16) $$MFI=\frac{600\times m}{t}$$
  where m is the mass of extruded polymer, t is time, and 600 is a conversion factor (10 min = 600 s).
➢ The filling time is the time taken to fill the mold cavity with molten polymer. This is defined in Equation (17):
  (17) $$t_{f}=\frac{L}{v}$$
  In this equation, $t_{f}$ denotes filling time, L denotes flow length, and v denotes flow velocity.
➢ The injection pressure calculates the molding force for producing the molded polymer. This is expressed in Equation (18):
  (18) $$P=\frac{12\eta LQ}{h^{3}W}$$
  Here, P indicates injection pressure, Q indicates volumetric flow rate, h indicates channel thickness, and w indicates channel width.

#### 5.2.5. FEA Metrics

FEA metrics are used to analyze a component’s response to simulated loading and operating conditions to assess its structural, thermal and deformation behavior.

➢ The Von Mises stress is the equivalent stress that can be used to predict yielding of a material under complex loading. This is expressed in Equation (19):
  (19) $$\sigma_{v}=\sqrt{3.J_{2}}$$
  where $\sigma_{v}$ denotes the Von Mises stress, and $J_{2}$ denotes the second invariant of the deviatoric stress tensor.
➢ Total deformation describes how much the component is displaced when a load is applied. This is represented in Equation (20):
  (20) $$\delta =\frac{FL}{AE}$$
  Here, δ represents deformation, F represents the applied force, L represents the original length, A represents the cross-sectional area, and E represents Young’s modulus.
➢ Temperature distribution describes the change in temperature in the component with time. This is defined in Equation (21):
  (21) $$\frac{\partial T}{\partial t}=\alpha \nabla^{2}T$$
  where $\frac{\partial T}{\partial t}$ denotes rate of change in temperature with respect to time, α denotes thermal diffusivity, and $\nabla^{2}T$ denotes the laplacian of temperature.

### 5.3. Results

The results show that PEEK has a high degree of thermal stability, mechanical strength and shear-thinning properties which are appropriate for high-performance applications. The Carreau–Yasuda fit with R^2^ = 0.97 provides a quantitative material-level basis for the subsequent flow analysis, while the simulated filling, pressure and FEA responses extend this evidence to the bearing component. The validation of flow behavior under processing conditions by the rheological analysis and model fitting is accurate. Efficient mold filling, uniform pressure distribution and optimized design performance are demonstrated with injection molding and CAD simulations. Finally, FEA and statistical validation ensure reliability, minimal deformation and high accuracy, even for practical engineering applications.

#### 5.3.1. Material Preparation Results

Pre-processing in this section includes heating the PEEK material to remove absorbed moisture prior to further processing. It guarantees process stability and ensures that the material is free of defects like voids, bubbles and degradation during high-temperature processing.

(A detailed breakdown of the PEEK drying process parameters is provided in Table S4 of the Supplementary Materials).

The drying process parameters of PEEK are displayed in Table S4, which displays the controlled thermal conditions to which PEEK needs to be subjected to remove moisture before processing. The findings have confirmed that the drying treatment process can successfully decrease the moisture content from 0.120% to 0.00099%; the moisture-free polymer is helpful for high-quality manufacturing application.

**Figure 4:**
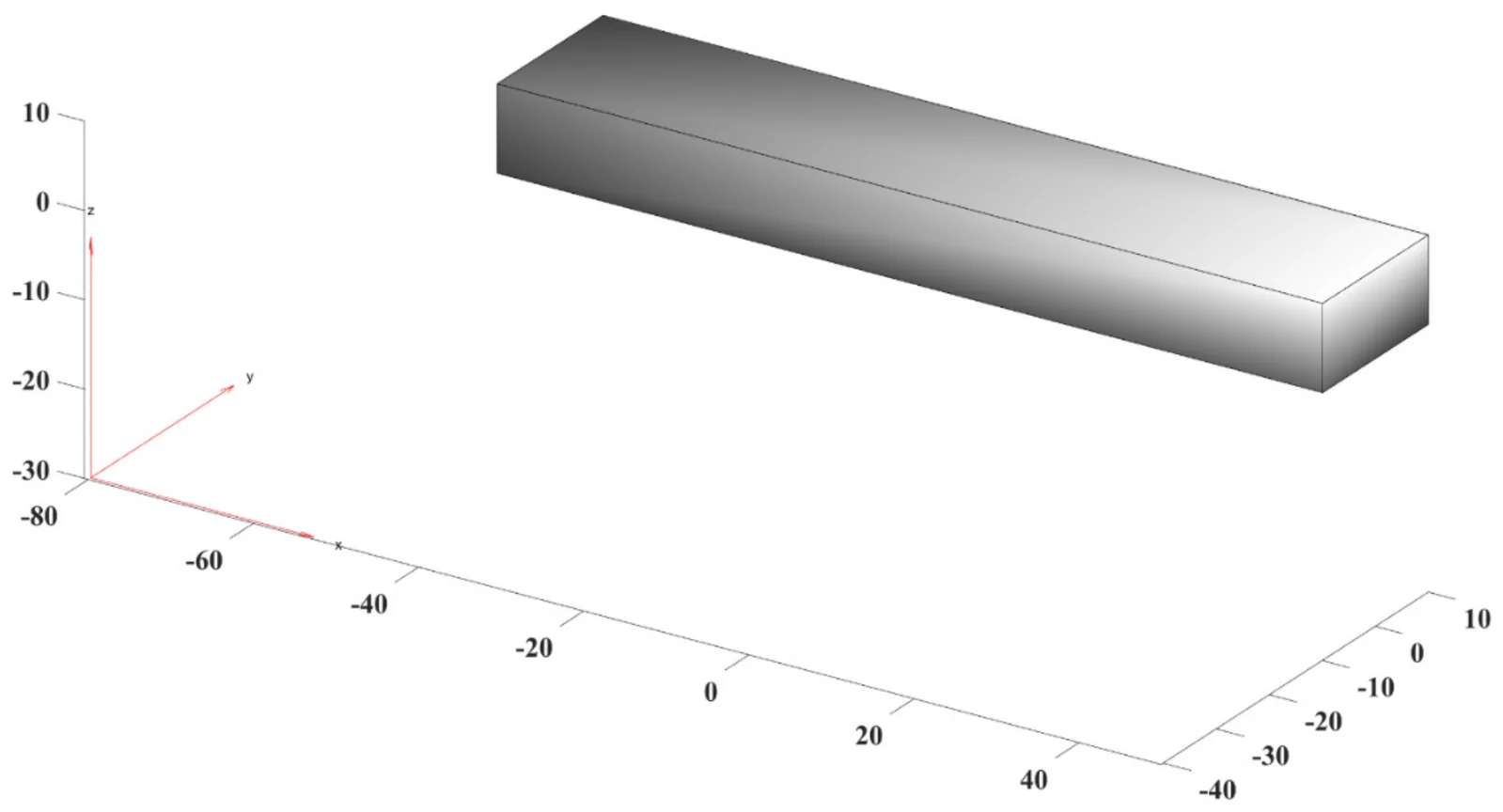
Moisture-free PEEK material after drying.

The moisture-free PEEK material that is poured after drying occurs is modeled as a 3D solid geometry, as seen in Figure 4. The uniform structure ensures defect-free material for further processing due to successful moisture removal. This is a validation of the efficiency of material drying in enhancing the quality and stability of the materials.

#### 5.3.2. Material Characterization Results

This section systematically evaluates the thermal, mechanical and physical properties of the selected polymer in order to understand its behavior under processing and service conditions. It offers fundamental input parameters like transition temperatures, strength parameters and properties of the structure needed for simulating and analyzing its performance correctly.

As can be seen in Table 8, PEEK has a high thermal stability, making it an ideal material for use at high temperatures. The results demonstrate the ability of PEEK to retain structural integrity, which is enabled by its high melting and degradation temperatures.

Table 9 shows a progressive reduction in viscosity with increasing temperature and shear rate, confirming the temperature-dependent shear-thinning behavior of the material.

(A detailed breakdown of the rheometer configuration and rheological measurement conditions is provided in Table S5 of the Supplementary Materials).

The rheological characterization of PEEK was performed using rotational and capillary rheometers to obtain viscosity data over the required temperature and shear rate ranges in Table S5.

(A detailed breakdown of the DSC and TGA test conditions and thermal characterization parameters is provided in Table S6 of the Supplementary Materials).

The DSC and TGA measurements were conducted under controlled thermal conditions, with the test parameters and principal thermal characteristics summarized in Table S6. The specified sample masses, temperature ranges, heating rates, nitrogen atmosphere and gas-flow conditions define the experimental conditions used for evaluating the thermal behavior of PEEK.

The material properties and geometric dimensions of the PEEK specimen for analysis are exhibited in detail in Table 10. The values reflect high mechanical properties and heat stability, suitable for structural and high-performance engineering applications.

**Figure 5:**
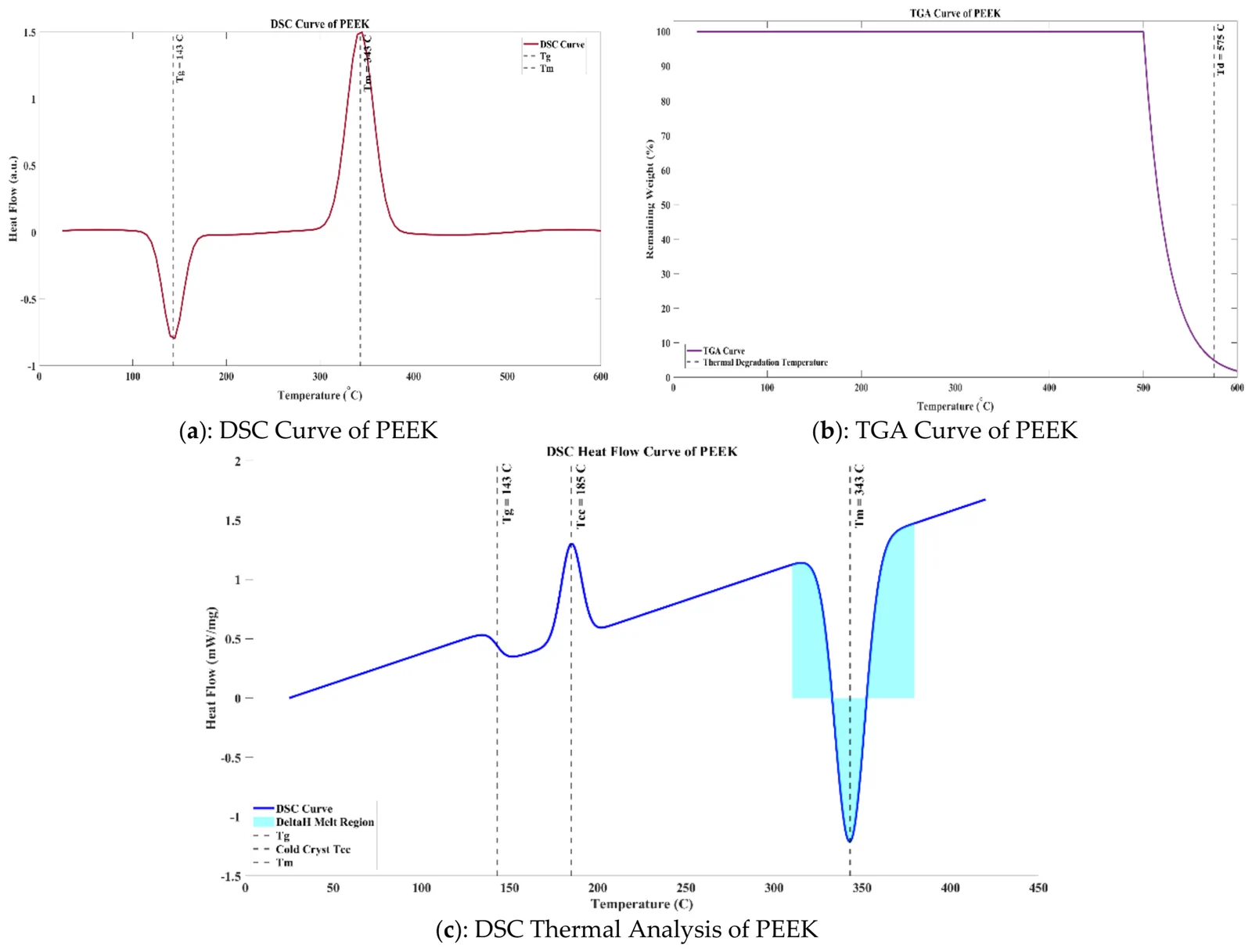
Thermal analysis of PEEK (DSC and TGA curves).

The DSC curve of PEEK (Figure 5a) depicts glass transitions and melting characteristics, and the melting temperature (Tm) is clearly visible as the endothermic peak. This confirms its thermal transition properties, showing that the material has a semi-crystalline nature. The thermal stability of PEEK is demonstrated in Figure 5b by the lack of weight loss at high temperatures, followed by an accelerated loss of weight. This means that the material has a high thermal resistance and is suitable for use in high-temperature applications. Figure 5c shows the DSC curve of PEEK, highlighting its glass transition at 143 °C, cold crystallization at 185 °C, and sharp melting peak at 343 °C.

#### 5.3.3. Rheological Testing Results

This section explains the deformation and flow behavior of PEEK, which are related to its viscoelastic properties (storage modulus, loss modulus, viscosity and shear response). This analysis enables us to understand the material’s flow behavior and processability in conditions such as injection molding.

The rheological properties of PEEK are given in Table 11, with the storage modulus indicating the elastic properties of the material and the loss modulus indicating the viscous properties. The frequency-dependent storage modulus (G′) and loss modulus (G″) are additionally presented over the investigated frequency range to show the evolution of the elastic and viscous contributions with oscillation frequency. The frequency response is discussed together with the reported average values to provide a more complete representation of the viscoelastic behavior of PEEK. The results show a balanced viscoelastic behavior, which is indicative of good energy storage and dissipation properties under deformation.

**Figure 6:**
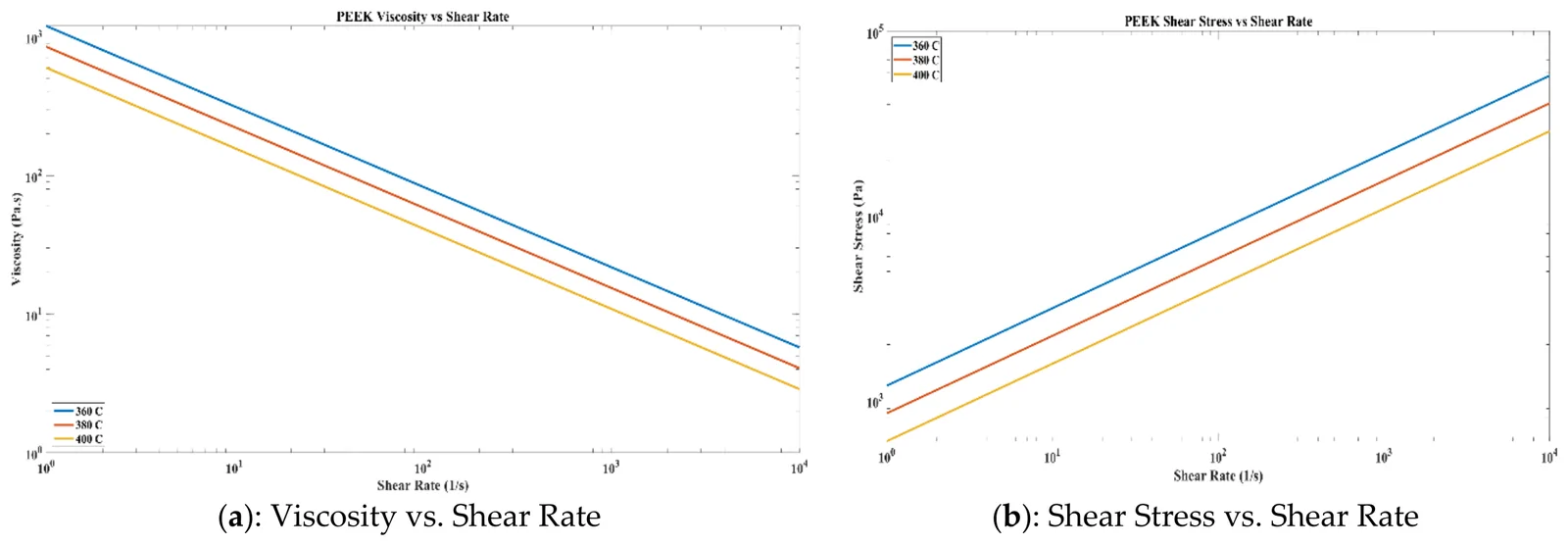
Rheological behavior of PEEK (viscosity and shear stress vs. shear rate).

As seen in Figure 6a, the viscosity curve has a downward trend for PEEK at different temperatures, which means that PEEK is shear-thinning at these temperatures. This means that the polymer melt has enhanced flowability during processing at high shear rates. The non-Newtonian flow behavior of PEEK is demonstrated in Figure 6b of the shear stress vs. shear rate curve, with a linear increase. This means that a higher shear rate would result in more stress for the processing, as this is significant for control.

#### 5.3.4. Rheological Model Fitting Results

This section compares the shear rate–viscosity relationship for the experimental data with the predictions of various constitutive models. This is useful for the identification of the model that best describes the non-linear flow behavior of the polymer melt under various processing conditions.

**Figure 7:**
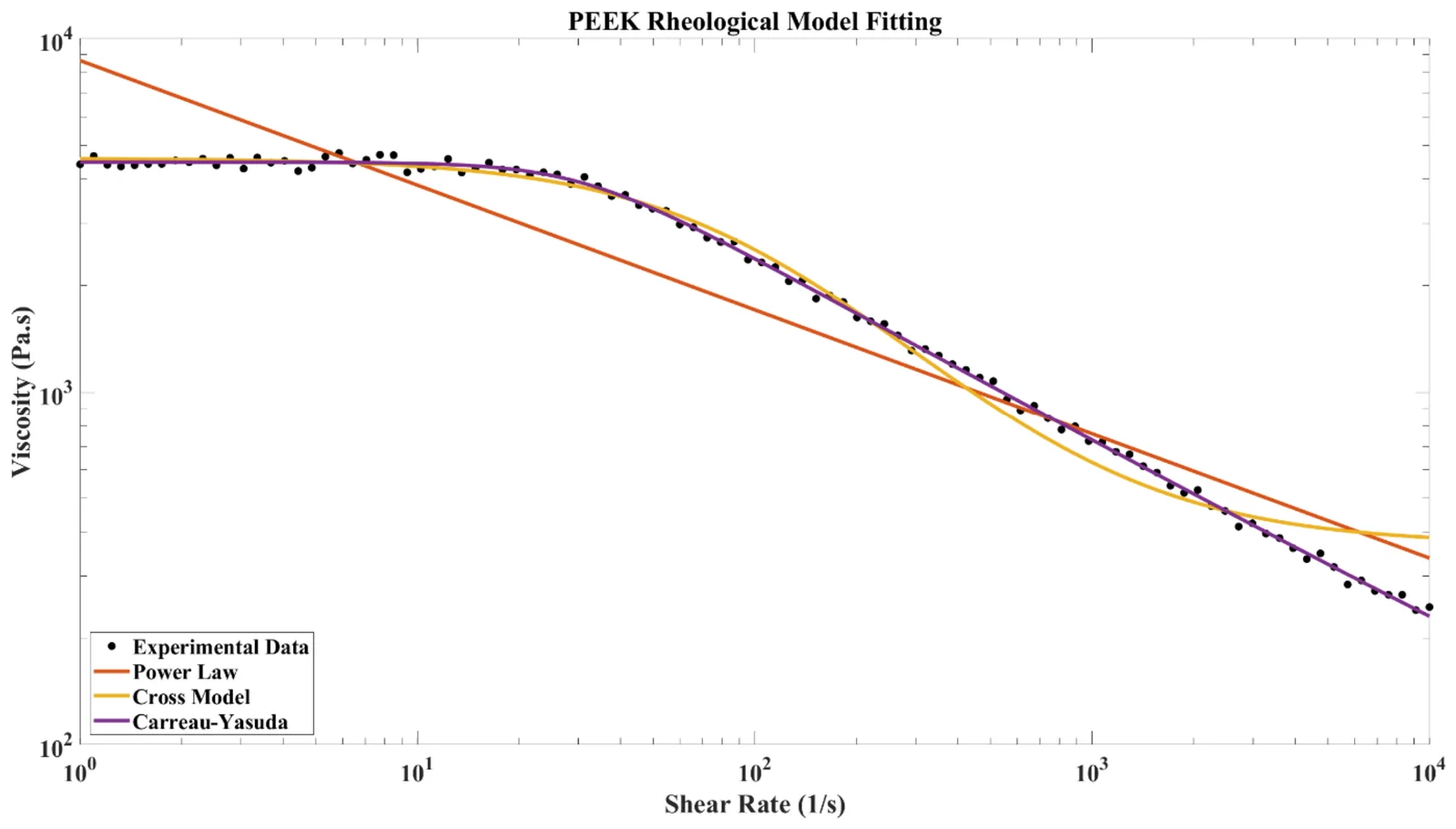
Rheological model fitting.

The experimental values and several models, such as the Power Law, Cross, and Carreau–Yasuda models, were compared over a wide shear rate range, as shown in Figure 7. The Carreau–Yasuda model is able to give a close prediction for the polymer flow behavior when compared to the experimental data and shows a higher accuracy in predicting the non-linear viscosity variation. The experimental identification of the constitutive representation most consistent with the measured PEEK response before its use in process-level interpretation represents an important contribution. The obtained R^2^ of 0.97 indicates that the Carreau–Yasuda formulation provides a substantially suitable representation of the measured non-linear viscosity behavior within the investigated conditions. The superior agreement of the Carreau–Yasuda formulation indicates that the measured PEEK response cannot be represented equally well by simpler constitutive descriptions across the investigated shear rate range. This finding is important because the constitutive choice directly determines the viscosity response supplied to the subsequent processing simulation and therefore affects predicted flow and pressure development.

**Figure 8:**
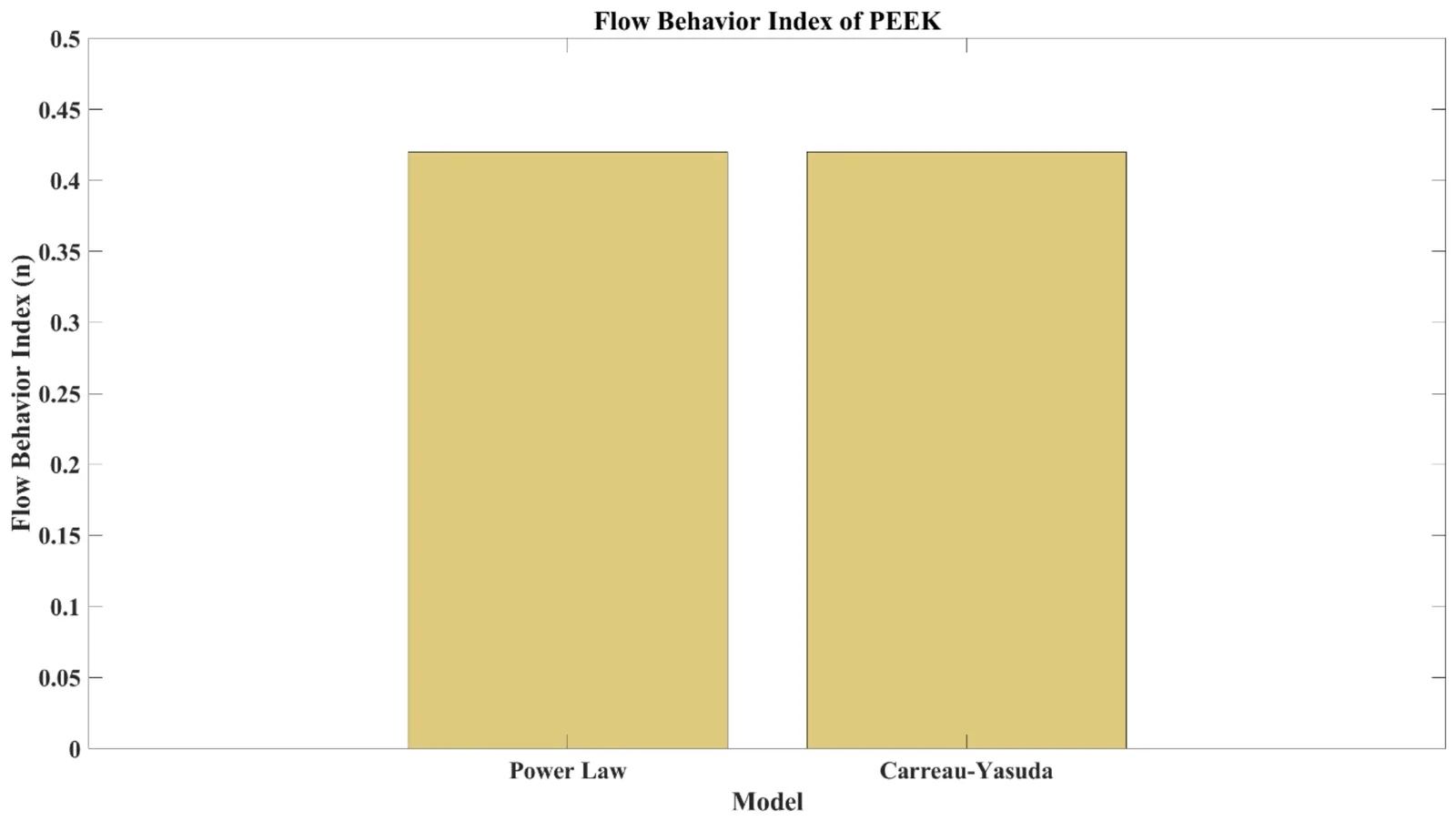
Flow behavior index.

Figure 8 shows the flow behavior index values for different models, which are less than 1, confirming the shear-thinning nature of PEEK. The consistency between the models indicates stable rheological behavior and the reliable prediction of processing performance.

Table 12 defines the melt temperature, mold temperature, injection pressure, filling time, and thermal boundary conditions used in the processing simulation, while the Carreau–Yasuda model was used to describe the material viscosity.

Table 13 presents the rheological parameters, processing conditions, material properties, and mechanical and thermal boundary conditions which were integrated to establish the numerical simulation model.

The experimentally obtained rheological, thermal, and mechanical parameters were incorporated into the numerical model to define the constitutive behavior, processing conditions, and thermo-mechanical properties shown in Table 14.

#### 5.3.5. Injection Molding Simulation Results

Melt flow, filling and pressure distribution were modeled in the cavity to determine the suitability of the material for efficient and defect-free processing.

**Figure 9:**
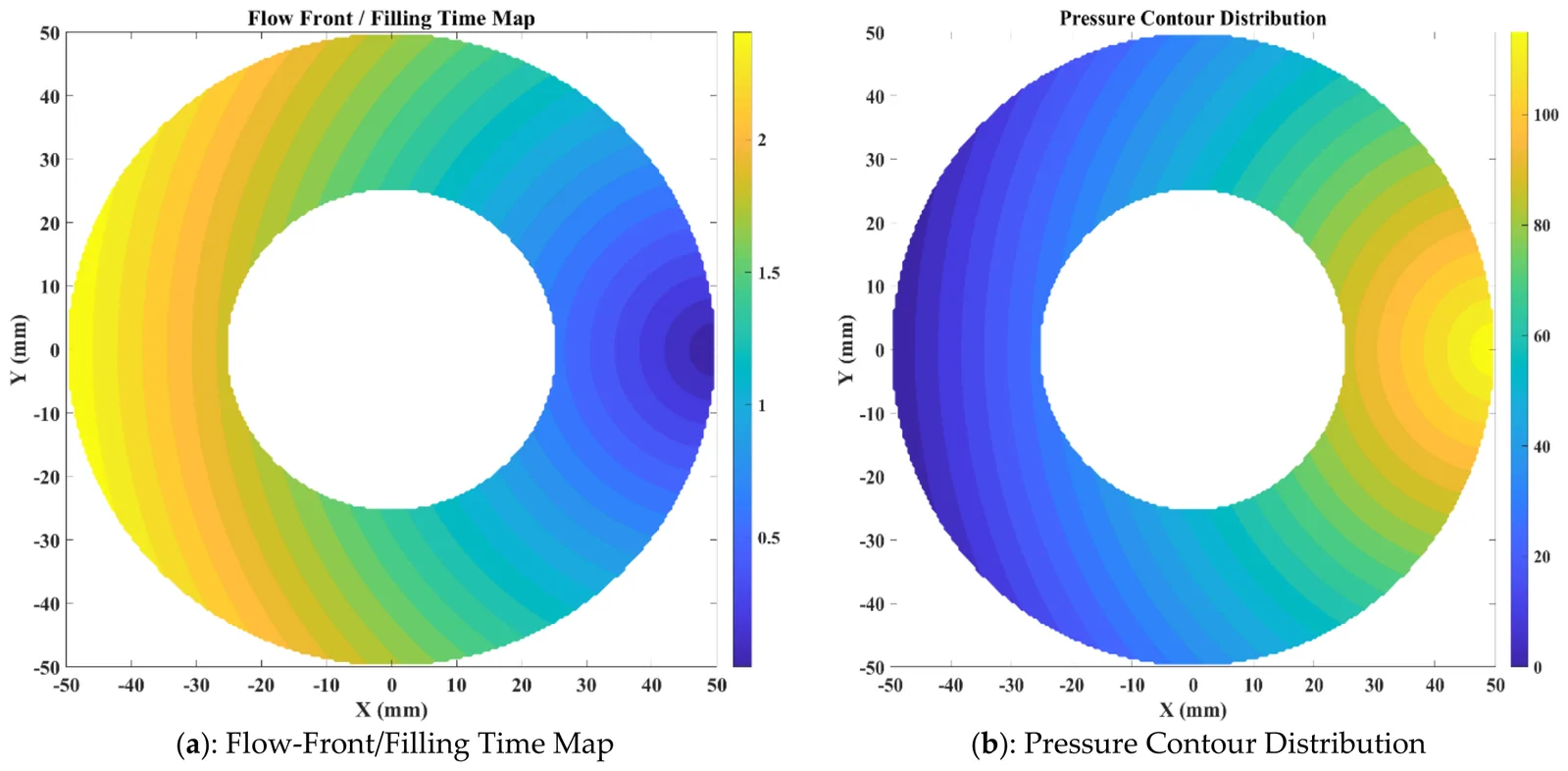
Flow-front and pressure distribution.

In the mold cavity, the flow-front and filling time distribution are displayed in Figure 9a, which represents the filling process of the molten PEEK. The uniform gradient shows efficient and complete filling behavior. The pressure contour distribution throughout the mold is shown in Figure 9b, which reveals the areas of high and low injection pressure. This is useful for determining critical zones and optimizing processing conditions to prevent defects. The relatively uniform flow-front progression indicates that the selected processing conditions provide consistent cavity filling without pronounced flow imbalance in the analyzed bearing geometry. The pressure distribution further identifies regions where localized pressure accumulation may influence defect formation, and therefore provides a direct process-level consequence of the selected material-flow description.

(A detailed breakdown of the gate design and injection molding process parameters is provided in Table S7 of the Supplementary Materials).

Table S7 shows that the simulation employed a single edge gate with a 4 mm gate width, 1.5 mm gate thickness and 2.5 mm gate length, together with an injection pressure of 80 MPa and melt and mold temperatures of 380 °C and 180 °C, respectively. Under these conditions, the predicted filling ratio was 0.96 with a fill imbalance of 0.04, while the maximum predicted warpage and stress were 0.05 mm and 6.85 MPa, respectively.

#### 5.3.6. CAD Modeling Results

This section describes a geometric representation and the meshed structure of the PEEK bearing to provide accurate material flow, stress distribution and thermal simulation.

**Figure 10:**
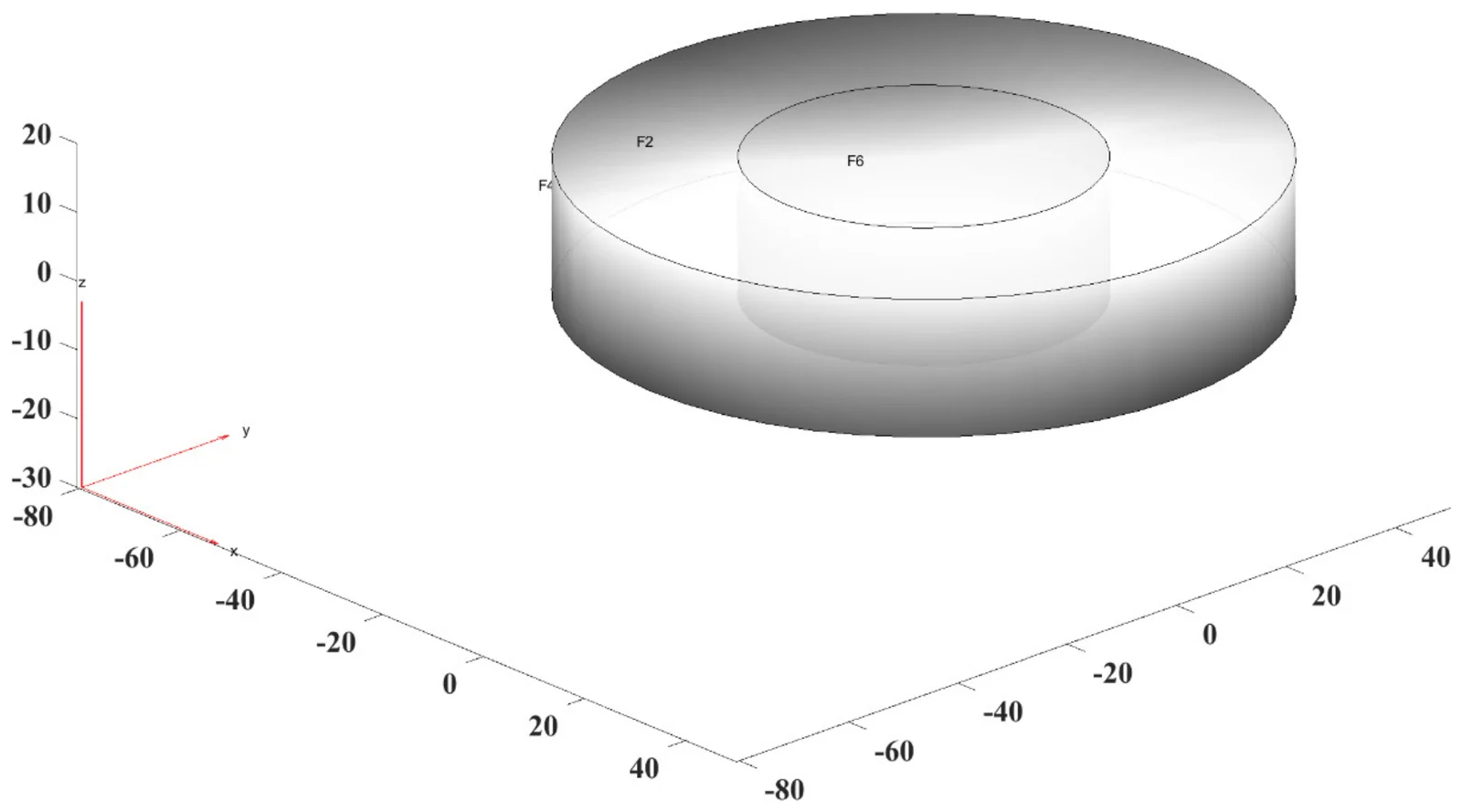
3D CAD model of PEEK bearing.

Figure 10 displays the 3D CAD model of an aerospace bearing made of PEEK, which has a cylindrical shape with two surfaces labeled F2 and F6 for orientation and analysis purposes. The CAD representation is included to identify the component geometry, analysis surfaces and coordinate reference used consistently in the subsequent injection molding and FEA analyses. The F2 and F6 surfaces provide geometric references for interpreting the predicted flow and structural responses rather than serving only as visual illustrations. The coordinate axes are used to give the spatial reference for the simulation, which is important for representing the flow and the stress distribution. This model is used as the basis of injection molding and FEA for assessing structural and thermal performance.

**Figure 11:**
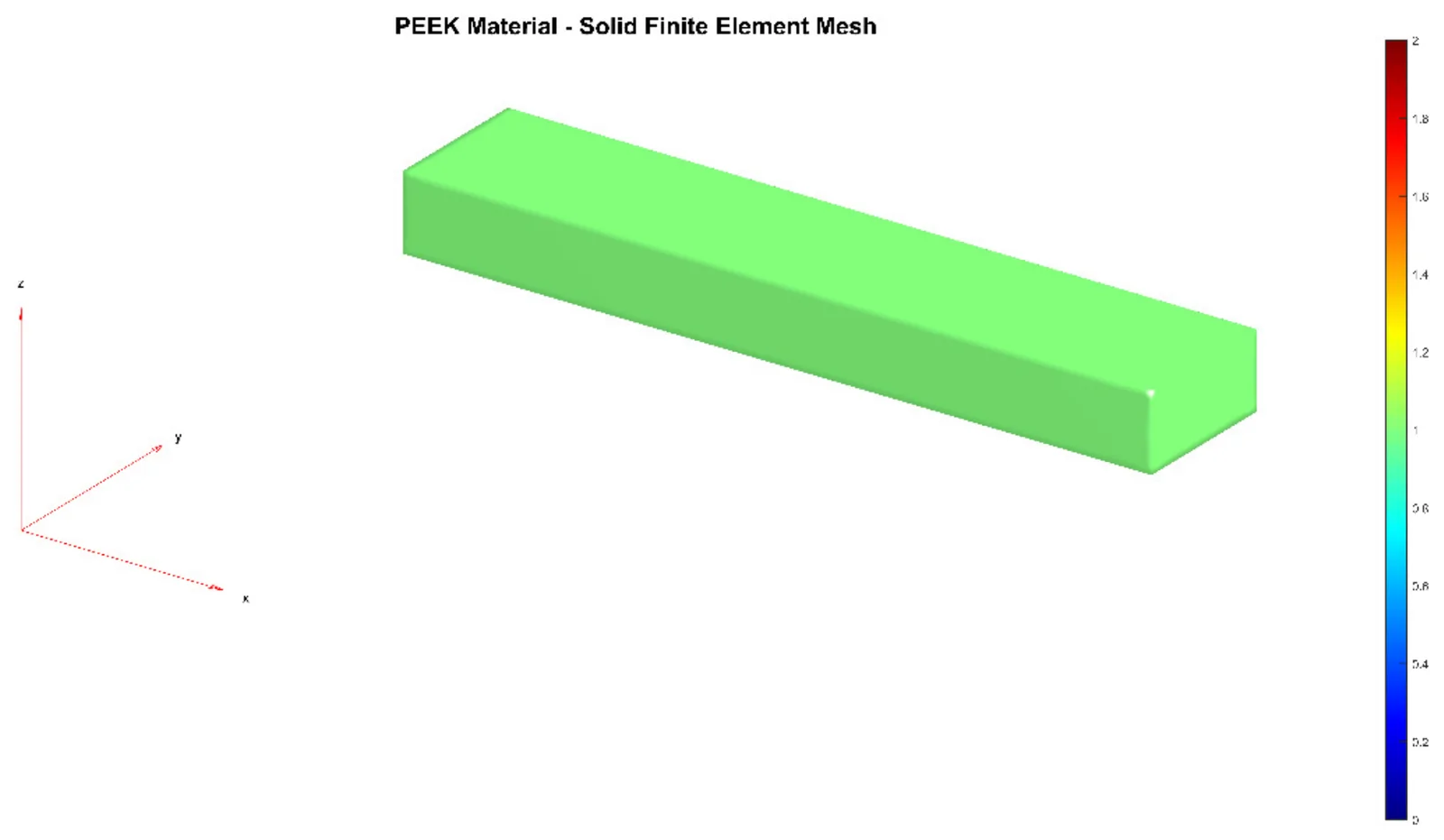
Finite element mesh structure.

The finite element mesh structure of the PEEK material is shown in Figure 11; the block is divided into elements to make it possible to simulate the stress and thermal behavior. The mesh representation is provided specifically to demonstrate the discretization used for calculating the stress, deformation and thermal fields reported in the FEA results. Its technical purpose is therefore to establish a numerical representation of the bearing geometry rather than to provide an additional visual description of the component. The color scale shows the variation in a physical property (such as stress or strain) from cell to cell in the mesh. This visualization is used as a basis for the correct FEA of PEEK parts in practical operating conditions. The 1.0 mm mesh was selected as the converged configuration because the stress and deformation changes decreased to 0.159% and 0.232%, respectively, in Table S8.

(A detailed breakdown of the mesh convergence cases and their stress and deformation results is provided in Table S8 of the Supplementary Materials).

(A detailed breakdown of the processing parameter ranges and optimized conditions is provided in Table S9 of the Supplementary Materials).

As shown in Table S9, the selected optimized conditions consisted of a melt temperature of 380 °C, mold temperature of 180 °C, injection pressure of 80 MPa, injection speed of 50 mm·s^−1^, holding pressure of 55 MPa and cooling time of 25 s. Under these conditions, the predicted dimensional deviation, warpage, maximum stress and deformation were 0.025 mm, 0.05 mm, 6.85 MPa and 0.019 mm, respectively.

#### 5.3.7. FEA Results

This section evaluates the structural, thermal and thermo-mechanical response of the PEEK bearing to determine stress distribution, deformation and the temperature change in its operation.

**Figure 12:**
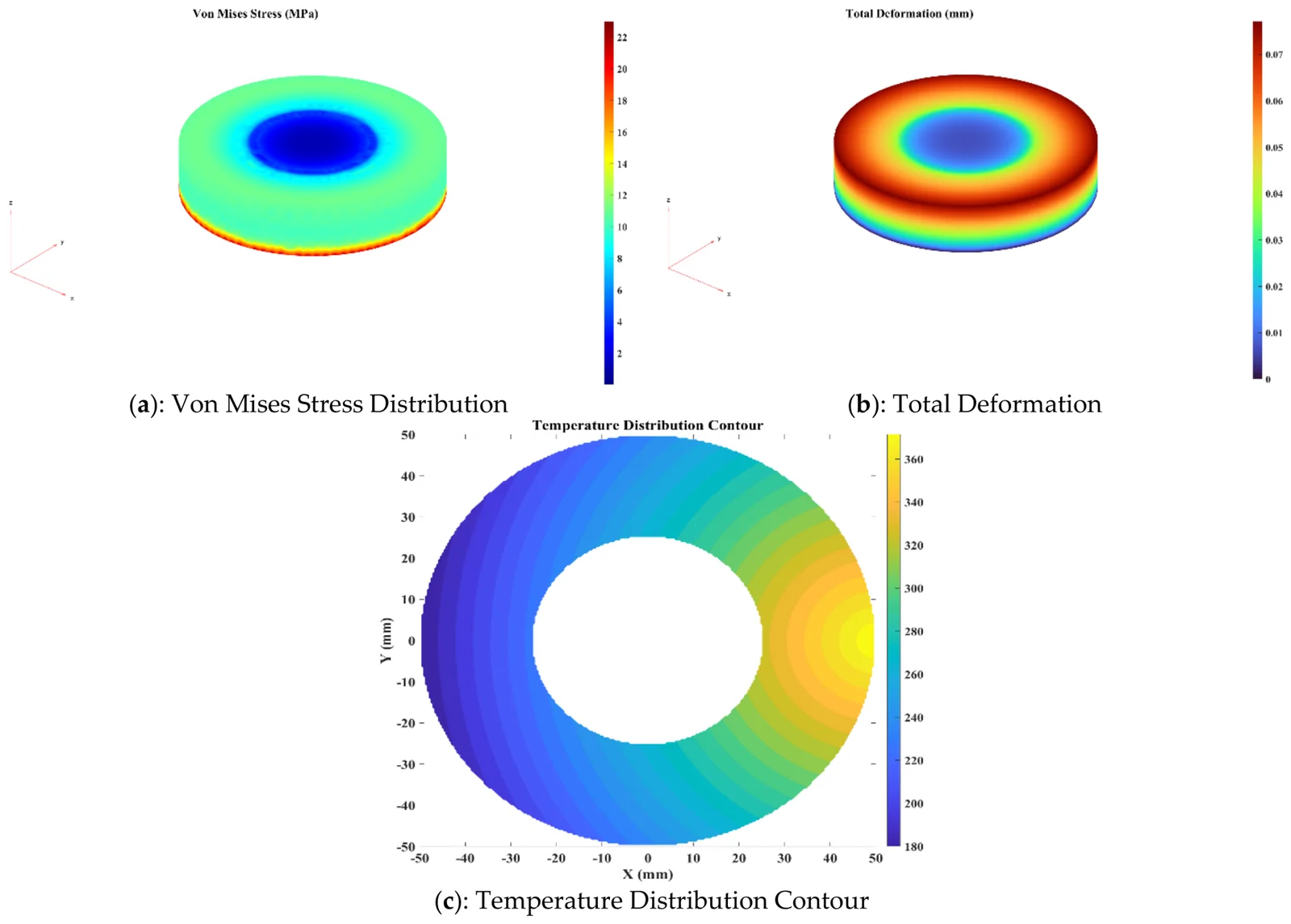
FEA results.

Figure 12a presents the Von Mises stress distribution throughout the component, with red indicating the areas of maximum stress concentration and blue indicating the areas of minimum stress. Unlike the geometric illustrations, Figure 12 provides direct simulation outputs that quantify the structural and thermal response of the PEEK bearing under the defined analysis conditions. The corresponding stress and deformation statistics provide numerical evidence beyond the color contours, including a mean stress of 7.5999 MPa and mean deformation of 0.025451 mm. The total deformation contour is shown in Figure 12b, with red areas corresponding to high deformation and blue areas to low deformation. The temperature distribution contour in Figure 12c depicts the variation in temperature within the structure, ranging from cooler blue areas to warmer yellow areas.

**Figure 13:**
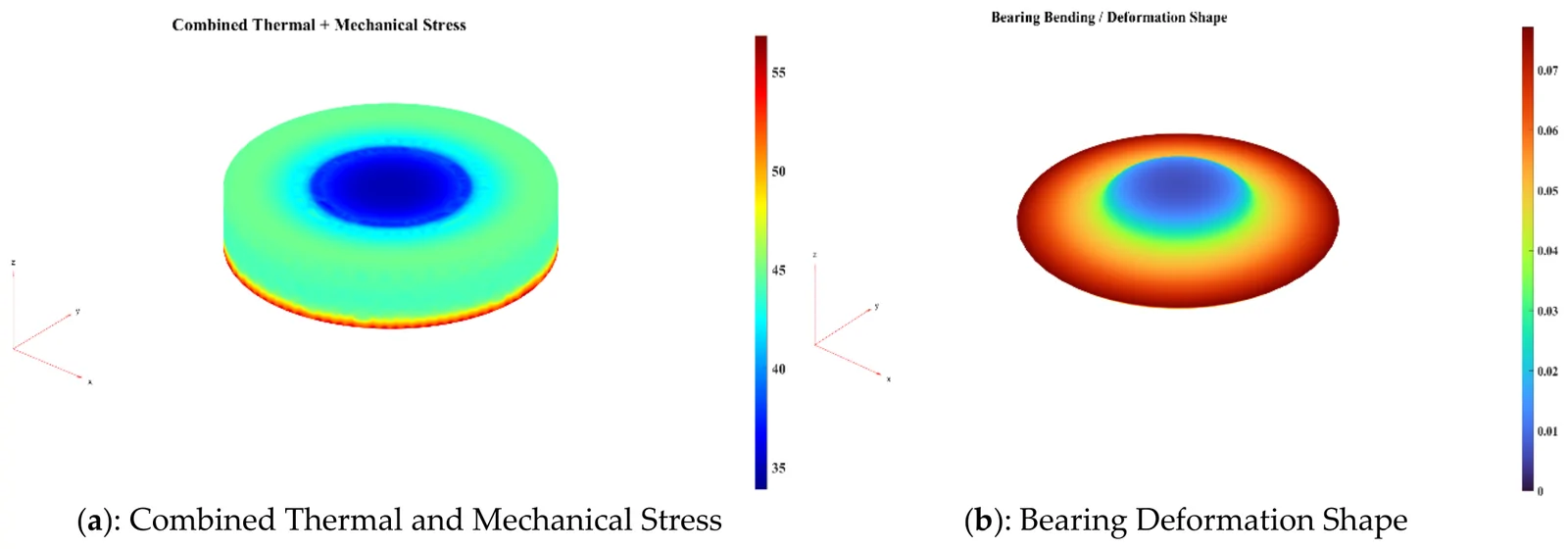
Thermo-mechanical stress and deformation analysis.

The combined thermal and mechanical stresses are displayed in Figure 13a, with red areas representing high stresses and blue areas representing low stresses of the bearing. Figure 13b illustrates the shape of the deformation of the bearings when under load, with red areas indicating maximum bending and blue areas indicating minimal deformation.

Table 15 summarizes the principal inputs and outputs used in the structural and thermo-mechanical assessment of the PEEK radial bearing, including the 5000 N applied radial load, fixed-support condition and operating temperature of 180 °C. Under these conditions, the calculated mean von Mises stress was 7.5999 MPa and the total deformation was 0.025451 mm, corresponding to an 8% stress utilization and an 87.4001 MPa stress margin relative to the reported tensile strength. The FEA model was defined using the loading, displacement constraints, contact conditions, material properties and thermal conditions summarized in Table 6. A uniform radial load of 5000 N was applied to the bearing inner surface, while the outer surface was fixed and the specified radial, axial and rotational constraints were imposed.

(A detailed breakdown of the FEA boundary conditions, material properties, and mesh characteristics is provided in Table S10 of the Supplementary Materials).

The final finite element model was generated using a quadratic tetrahedral mesh, and the principal mesh characteristics and quality indicators are summarized in Table S10.

(A detailed breakdown of the mesh parameters used in the convergence check is provided in Table S11 of the Supplementary Materials).

The 1.0 mm mesh was selected in Table S11 as the converged configuration because the stress and deformation changes decreased to 0.159% and 0.232%, respectively.

The material properties and thermo-mechanical parameters defined in Table 16 were subsequently used for mesh generation, loading application, and numerical FEA simulation.

(A detailed breakdown of the PEEK material specification and characterization conditions is provided in Table S12 of the Supplementary Materials).

The specified PEEK grade, drying conditions, initial and final moisture contents, and DSC/TGA measurement conditions were used consistently throughout the subsequent material characterization, and are shown in Table S12.

In Table 17, the numerical simulation results are compared with the corresponding experimental measurements to quantitatively assess the predictive accuracy of the proposed model.

#### 5.3.8. Statistical Validation

The statistical validation assesses the accuracy and reliability of the predicted results by comparing the predicted results with the expected results based on error metrics and correlation measures.

Table 18 shows the statistical analysis of stress and deformation; the mean value represents the overall performance, and the deviation from the mean value represents the variation in the results. Therefore, this is retained as a key quantitative result because it reports prediction error and correlation measures rather than repeating descriptive information presented elsewhere. The stress and deformation RMSE, MAE and R^2^ values provide a compact numerical assessment of prediction consistency. The low RMSE and MAE values confirm the high prediction accuracy of the model. Strong correlation and excellent model reliability are shown, with high R^2^ values (0.97). The statistical assessment provides quantitative evidence of agreement between the evaluated predicted and reference responses, with RMSE, MAE and R^2^ jointly indicating low prediction error and strong correspondence. Nevertheless, these metrics should be interpreted as computational validation rather than a substitute for direct experimental validation of the simulated bearing responses under physical injection molding conditions.

Table 19 shows that the investigated PEEK material maintained favorable thermal and mechanical characteristics, with a glass transition temperature of 143 °C, melting temperature of 343 °C, thermal degradation temperature of 575 °C, Young’s modulus of 3.6 GPa, and tensile strength of 95 MPa.

(A detailed breakdown of the validation dataset configuration and statistical evaluation criteria is provided in Table S13 of the Supplementary Materials).

An independent FEA validation dataset was used to evaluate the predictive performance of the numerical model for von Mises stress and total deformation, and this dataset is shown in Table S13.

(A detailed breakdown of the experimental–numerical validation results is provided in Table S14 of the Supplementary Materials).

The validation results demonstrated good agreement between the experimental measurements and numerical predictions, with R^2^ values ranging from 0.952 to 0.962, as shown in Table S14.

(A detailed breakdown of the comparative evaluation against reported approaches is provided in Table S15 of the Supplementary Materials).

Table S15 indicates that the proposed integrated framework achieved the lowest reported RMSE and MAE values of 0.482 and 0.351, respectively, while providing an R^2^ value of 0.958 across the evaluated comparison. In contrast, the rheology-only and FEA-only approaches provided narrower analytical coverage, whereas the integrated approaches incorporated multiple stages of material and process analysis.

(A detailed breakdown of the numerical predictions and reference experimental values is provided in Table S16 of the Supplementary Materials).

The numerical predictions shown in Table S16 showed a strong agreement with the reference values, with the maximum relative error limited to 2.19%, confirming the validity of the computational model.

### 5.4. Discussion

The results have shown that the proposed framework was able to capture the rheological behavior, thermal stability, and mechanical performance of PEEK, which are suitable for application in high-performance engineering fields. The key scientific findings are therefore concentrated in the experimentally measured rheological response, the comparative constitutive-model performance, the predicted filling and pressure behavior, and the quantified thermo-mechanical response. Beyond confirming the established properties of PEEK, the analysis shows that the experimentally observed shear-thinning response and constitutive-model selection provide a direct material basis for interpreting the subsequent processing and structural predictions. The principal finding is therefore the quantitative continuity from measured rheological behavior through simulated filling and pressure development to predicted thermo-mechanical response, rather than the individual confirmation of well-known PEEK properties. Moisture-free conditions for the material preparation stage would definitely improve the quality of processing and help to avoid defects. Thermal and mechanical characterization was also performed and showed that PEEK has a high stability and strength, and is thus suitable for an application as a bearing in the aerospace sector. The Carreau–Yasuda model is used to accurately predict the non-linear flow properties, with strong shear-thinning behavior revealed by rheological testing and model fitting, which leads to reliable processing insights. The established theoretical equations and characterization procedures are therefore treated as analytical tools, while the research contribution arises from their application to the experimentally characterized PEEK bearing system. The uniform flow-front distribution, together with the identified pressure variations, indicates that the selected processing conditions provide effective cavity filling while highlighting regions requiring process control. Injection molding simulation reveals uniform flow and optimized pressure distribution, which show the efficient molding of the mold and the reduction in the formation of defects. CAD modeling and FEA results provide an understanding of structural integrity and where stress, deformation and temperature distributions are within acceptable limits, ensuring the reliability of the component being used under operational conditions. Finally, the statistical validation, indicated by high R^2^ values and low error measures, ensures the accuracy and robustness of the proposed approach.

These existing studies (Gul et al. [14] and Herzog et al. [24]) showed that the thermal conductivity of the material was enhanced, and the thermo-mechanical behavior was predicted with good accuracy by using extrusion-based processing and surrogate modeling techniques, though with high processing complexity and limited validation in real manufacturing scenarios. In contrast, the proposed framework incorporates advanced experimental characterization, experimental rheological modeling, injection molding simulation, CAD-based design and FEA into a single streamline approach that subsequently allows for better prediction of flow, structure, and thermal properties. The contribution should therefore not be interpreted as a claim that coupling these computational tools is unprecedented, since similar integrated capabilities are established in polymer engineering practice. Instead, the research value lies in connecting experimentally measured PEEK rheology and comparative constitutive-model selection with the predicted behavior of the investigated aircraft bearing geometry. This is a combination of two things which makes the system more reliable, reduces the number of processing defects, and also gives a complete solution with a scalable model for practical industrial applications, outperforming the limitations found in the existing works. The research contribution is consequently positioned as a quantitative material-to-component analysis rather than as a simple combination of established software procedures. In particular, comparative rheological fitting identifies the constitutive representation used to interpret the processing response, while the subsequent simulations quantify how that material behavior manifests in filling, pressure, stress and deformation.

#### Limitations

- There was limited experimental validation under practical industrial manufacturing conditions.
- In particular, the simulated filling patterns, pressure fields, stress distributions and deformation responses were not directly compared with measurements obtained from physically molded PEEK bearing specimens.
- This study is computationally dependent on simulations, which results in the need for more time and resources.

## 6. Conclusions

This study successfully investigates the rheological behavior and processing characteristics of PEEK for high-performance aircraft bearing applications through a novel integrated experimental–computational framework. The proposed methodology uniquely combines four key components—material characterization, rheological testing with multi-model fitting, injection molding simulation, and multiphysics FEA—into a coupled predictive approach that bridges the critical gap between laboratory-scale material analysis and industrial-scale processing optimization. The principal scientific outcome is the establishment of a continuous quantitative pathway from experimentally measured PEEK rheology to constitutive-model selection and subsequently to process and thermo-mechanical predictions for an aircraft bearing geometry.

The major findings and novel contributions of this work are summarized as follows:

1. Comprehensive material characterization confirmed PEEK’s exceptional thermal stability (Tg = 143 °C, Tm = 343 °C, Td = 575 °C) and mechanical strength (tensile strength = 95 MPa, elastic modulus = 3.60 GPa), validating its suitability for demanding aerospace bearing applications.
2. Rheological testing revealed pronounced shear-thinning behavior, with viscosity decreasing significantly with increasing shear rate across all tested temperatures, demonstrating enhanced processability under high-shear injection molding conditions.
3. Comparative rheological model fitting established the Carreau–Yasuda model as the most accurate predictor of PEEK’s non-linear flow behavior (R^2^ = 0.97), outperforming Power Law and Cross models, providing a robust mathematical foundation for processing simulation.
4. The key novelty of this work—the integrated rheology–simulation framework—enabled the accurate prediction of mold filling behavior, pressure distribution, and temperature profiles during injection molding, with simulation results confirming homogeneous flow patterns and optimized processing conditions.
5. Multiphysics FEA incorporating thermo-mechanical coupling (σ = E(ε − αΔT)) provided comprehensive assessment of bearing performance under operational loads, with von Mises stress analysis and deformation patterns remaining within acceptable limits for aerospace applications.
6. Rigorous statistical validation confirmed the reliability of the proposed framework, with low error metrics (RMSE: 0.6854 MPa for stress, 0.003220 mm for deformation; MAE: 0.4850 MPa for stress, 0.002037 mm for deformation) and high correlation coefficients (R^2^ = 0.97), demonstrating excellent predictive accuracy.

The significant novelty of this work compared to existing literature lies in the following: (a) The seamless integration of experimental rheological characterization with advanced multiphysics simulation creates a truly coupled material–process–performance prediction platform. The novelty is consequently positioned in the material-specific quantitative linkage rather than in the generic integration of established simulation technologies. The framework traces experimentally measured PEEK rheology through constitutive-model comparison and selection to filling, pressure, stress and deformation predictions for the aircraft bearing configuration. (b) The application of comprehensive model comparison identifies the optimal rheological model for PEEK under injection molding conditions. (c) The thermo-mechanical coupling in FEA captures the combined effects of thermal expansion and mechanical loading on bearing performance. (d) The quantitative statistical validation establishes the framework’s reliability and transferability to other high-performance polymer systems.

The proposed framework has practical implications in industrial manufacturing, including less trial-and-error in tuning processing parameters, higher manufacturing efficiencies due to fewer defects through the prediction of possible defects, and improved part quality and reliability for aerospace applications; the approach can be expanded for other high-performance polymers or with complex geometries.

### Future Work

While this study establishes a robust foundation for integrated polymer processing analysis, several promising directions for future research emerge:

- Advanced Material Development: Investigate PEEK-based hybrid polymers and nanocomposites incorporating functional fillers (e.g., carbon nanotubes, graphene, hexagonal boron nitride) to enhance thermal conductivity, mechanical strength, and multifunctional performance for next-generation aerospace applications.
- Artificial Intelligence Integration: Introduce AI/ML-based predictive models (using neural networks or support vector regression (SVR)) for accelerated rheological parameter prediction, on-line processing condition optimization and reduced computation cost of multiphysics simulations.
- Process Optimization: The developed framework can further be extended in optimizing injection molding parameters (melt temperature, mold temperature, injection pressure and cooling rate) to multiple industrial applications for products such as automotive, biomedical and electronic components, in addition to bearings.
- Multi-Material Systems: Expand the methodology to multi-material and functionally graded PEEK components, addressing the growing demand for tailored property distributions in advanced engineering structures.
- Long-Term Performance Assessment: Integrate fatigue analysis, creep behavior and environmental degradations such as moisture absorption, UV exposure and chemical resistance into FEA simulation for long-term durability and service life prediction of PEEK components under actual operating conditions.
- Experimental Validation Under Industrial Conditions: Conduct detailed experimental trials with actual manufacturing conditions to further validate the predictive capabilities of the proposed framework and make it robust for industrial use.
- Sustainability Integration: Consider environmentally responsible PEEK processing routes: recycling, bio-based precursors, and less energy-demanding manufacture. Meet sustainability goals while maintaining high-performance characteristics.

In summary, this work delivers a comprehensive, validated, and industrially relevant framework for high-performance polymer processing, significantly advancing the state of the art in integrated experimental–computational materials engineering. The demonstrated methodology produces immediate benefits for the manufacture of PEEK bearings and could be translated as a platform for application across other advanced polymer processing challenges and designs.

## Figures and Tables

**Table 1:** Experimental characterization conditions, material properties and rheological model validation parameters for PEEK.

Characterization	Parameter	Reported/Revised Value	Unit/Description
Rheological Testing	Rheometer configuration	Rotational rheometer	Instrument configuration
Rheological Testing	Rheometer geometry	Parallel-plate	Geometry
Rheological Testing	Plate diameter	25	mm
Rheological Testing	Plate gap	1	mm
Rheological Testing	Measurement mode	Steady-shear viscosity	Rheological mode
Rheological Testing	Supplementary mode	Oscillatory shear	Viscoelastic characterization
Rheological Testing	Oscillatory strain amplitude	5	%
Rheological Testing	Frequency range	0.1–100	rad/s
Rheological Testing	Shear rate range	1–3000	s^−1^
Rheological Testing	Test temperatures	360, 370, 380, 390, 400	°C
Rheological Testing	Temperature stabilization time	10	min
Rheological Testing	Storage modulus, G′	226,368.5	Pa
Rheological Testing	Loss modulus, G″	105,018.4	Pa
DSC Analysis	Analysis technique	Differential Scanning Calorimetry	Thermal characterization
DSC Analysis	Sample mass	8	mg
DSC Analysis	Atmosphere	Nitrogen	Inert atmosphere
DSC Analysis	Nitrogen flow rate	50	mL/min
DSC Analysis	Initial temperature	25	°C
DSC Analysis	Final temperature	400	°C
DSC Analysis	Heating rate	10	°C/min
DSC Analysis	Glass transition temperature, Tg	143	°C
DSC Analysis	Melting temperature, Tm	343	°C
TGA	Analysis technique	Thermogravimetric Analysis	Thermal-stability characterization
TGA	Sample mass	10	mg
TGA	Atmosphere	Nitrogen	Inert atmosphere
TGA	Nitrogen flow rate	50	mL/min
TGA	Initial temperature	30	°C
TGA	Final temperature	800	°C
TGA	Heating rate	10	°C/min
TGA	Degradation temperature, Td	575	°C
Tensile Testing	Testing standard	ASTM D638	Tensile-test standard
Tensile Testing	Specimen geometry	Type I	ASTM specimen
Tensile Testing	Gauge length	50	mm
Tensile Testing	Crosshead speed	5	mm/min
Tensile Testing	Test temperature	23 ± 2	°C
Tensile Testing	Number of specimens	5	Specimens
Tensile Testing	Tensile strength	95	MPa
Tensile Testing	Young’s modulus	3.6	GPa
Tensile Testing	Data reporting	Mean of five specimens	Statistical reporting
Model Validation	Rheological model	Carreau–Yasuda	Constitutive model
Model Validation	Coefficient of determination	0.97	R^2^
Model Validation	Model fitting criterion	Non-linear least-squares fitting	Fitting method

**Table 2:** Fitted rheological model parameters and statistical diagnostic results.

Model Parameter/Diagnostic	Symbol	Fitted Value	95% Confidence Interval—Lower	95% Confidence Interval—Upper	Standard Error	Unit
Zero-shear viscosity	η_0_	4200	4015.2	4384.8	94.29	Pa·s
Infinite-shear viscosity	η^∞^	0.12	0.108	0.132	0.0061	Pa·s
Relaxation time	λ	0.018	0.01674	0.01926	0.00064	s
Flow behavior index	n	0.62	0.606	0.634	0.00714	Dimensionless
Yasuda parameter	a	2	1.84	2.16	0.0816	Dimensionless
Coefficient of determination	R^2^	0.97	0.9638	0.9762	0.00316	Dimensionless
Root mean square error	RMSE	0.6854	0.6461	0.7247	0.0201	MPa
Mean absolute error	MAE	0.485	0.4529	0.5171	0.0164	MPa
Residual mean	ē	0.0021	−0.0184	0.0226	0.0105	MPa
Residual standard deviation	SDres	0.6849	0.646	0.7238	0.0198	MPa
Maximum absolute residual	|e|max	1.842	1.7015	1.9825	0.0717	MPa
Residual normality *p*-value	*p*	0.184	0.126	0.242	0.0296	Dimensionless
Residual autocorrelation	ρ_1_	0.031	−0.021	0.083	0.0265	Dimensionless
Number of rheological observations	N	60	60	60	0	Observations

**Table 3:** DSC-based melting enthalpy and crystallinity parameters of PEEK.

Parameter	Symbol	Numerical Value	Unit/Description
Material	M	PEEK	Polymer
Measured melting enthalpy	ΔHm	72	J·g^−1^
Theoretical melting enthalpy at 100% crystallinity	ΔHm^0^	130	J·g^−1^
Reference basis	Ref	100% crystalline PEEK	Theoretical reference
DSC heating rate	β	10	°C·min^−1^
DSC atmosphere	A	Nitrogen	Inert atmosphere
DSC sample mass	m	8.5	mg
Crystallinity	Xc	55.385	%
Crystallinity fraction	Xc,f	0.55385	Dimensionless
Cold-crystallization enthalpy	ΔHcc	0	J·g^−1^
Normalized melting enthalpy	ΔHm,n	72	J·g^−1^
Calculation method	Method	DSC enthalpy normalization	Method

**Table 4:** Thermo-mechanical material properties and simulation parameters of PEEK.

Material Characteristic	Symbol	Numerical Value	Unit
Material	M	PEEK	Dimensionless
Density	ρ	1320	kg·m^−3^
Young’s modulus	E	3.6	GPa
Poisson’s ratio	ν	0.38	Dimensionless
Tensile yield strength	σy	95	MPa
Ultimate tensile strength	σUTS	100	MPa
Tensile strain at yield	εy	0.03	mm/mm
Flexural modulus	Ef	3.7	GPa
Flexural strength	σf	165	MPa
Compressive strength	σc	110	MPa
Shear modulus	G	1.3043	GPa
Thermal conductivity	k	0.25	W·m^−1^·K^−1^
Specific heat capacity	Cp	1300	J·kg^−1^·K^−1^
Coefficient of thermal expansion	α	47.000 × 10^−6^	K^−1^
Glass transition temperature	Tg	143	°C
Melting temperature	Tm	343	°C
Continuous service temperature	Tservice	180	°C
Reference temperature	Tref	25	°C
Operating temperature	Top	180	°C
Temperature difference	ΔT	155	°C
Thermal strain	εth	0.007285	Dimensionless
Applied radial load	F	5000	N
Mean von Mises stress	σVM	7.5999	MPa
Total deformation	U	0.025451	mm
Stress utilization	Uσ	8	%
Stress safety margin	Mσ	87.4001	MPa
Stress RMSE	RMSEσ	0.6854	MPa
Stress MAE	MAEσ	0.485	MPa
Deformation RMSE	RMSEU	0.00322	mm
Deformation MAE	MAEU	0.002037	mm
Coefficient of determination	R^2^	0.97	Dimensionless

**Table 5:** Thermo-mechanical FEA parameters and coupled response.

Parameter	Symbol	Numerical Value	Unit
Reference temperature	Tref	25	°C
Operating temperature	Top	180	°C
Temperature difference	ΔT	155	°C
Young’s modulus	E	3.6	GPa
Poisson’s ratio	ν	0.38	Dimensionless
Thermal conductivity	k	0.25	W·m^−1^·K^−1^
Specific heat capacity	Cp	1300	J·kg^−1^·K^−1^
Density	ρ	1320	kg·m^−3^
Thermal expansion coefficient	α	47.000 × 10^−6^	K^−1^
Applied radial load	F	5000	N
Load direction	θ	0	°
Thermal strain	εth	0.007285	Dimensionless
Thermal expansion per unit length	εth × 100	0.7285	%
Thermo-mechanical coupling factor	Kth	1	Dimensionless
Mechanical stress	σVM	7.5999	MPa
Total deformation	U	0.025451	mm
Stress utilization	Uσ	8	%
Stress safety margin	Mσ	87.4001	MPa
Thermal conductivity contribution	Qk	0.25	W·m^−1^·K^−1^
Thermal expansion contribution	Qα	0.007285	Dimensionless
Coupled response indicator	Cth	0.05828	Dimensionless
Stress RMSE	RMSEσ	0.6854	MPa
Stress MAE	MAEσ	0.485	MPa
Deformation RMSE	RMSEU	0.00322	mm
Deformation MAE	MAEU	0.002037	mm
Coefficient of determination	R^2^	0.97	Dimensionless

**Table 6:** Initial and final moisture content measurement and uncertainty analysis.

Measurement Parameter	Symbol	Initial Condition	Final Condition	Unit
Moisture content	M	0.12	0.00099	wt.%
Measurement technique	MT	Karl Fischer titration	Karl Fischer titration	Method
Measurement principle	MP	Coulometric water determination	Coulometric water determination	Method
Measurement instrument	MI	Karl Fischer moisture analyzer	Karl Fischer moisture analyzer	Instrument
Instrument resolution	R	0.0001	0.0001	wt.%
Instrument accuracy	A	±0.00100	±0.00100	wt.%
Measurement uncertainty	U	±0.00200	±0.00020	wt.%
Number of measurements	n	5	5	Measurements
Mean moisture content	M̄	0.12	0.00099	wt.%
Standard deviation	SD	0.0012	0.00008	wt.%
Standard error	SE	0.00054	0.00004	wt.%
Relative uncertainty	UR	1.667	20.202	%
Moisture reduction	ΔM	0.11901	0.11901	wt.%
Moisture reduction percentage	Rm	99.175	99.175	%

**Table 7:** Repeatability and statistical analysis of processing and numerical responses.

Response Parameter	Unit	Mean Value	Standard Deviation	Standard Error	95% CI Lower	95% CI Upper	CV (%)	Repeatability (%)
Applied radial load	N	5000	18.5	5.84	4987.02	5012.98	0.37	99.63
Operating temperature	°C	180	1.2	0.38	179.16	180.84	0.667	99.33
Melt temperature	°C	380	2.1	0.66	378.53	381.47	0.553	99.45
Mold temperature	°C	180	1.5	0.47	178.95	181.05	0.833	99.17
Injection pressure	MPa	80	1.1	0.35	79.23	80.77	1.375	98.63
Filling time	s	1.85	0.031	0.0098	1.8283	1.8717	1.676	98.32
Von Mises stress	MPa	7.5999	0.084	0.0265	7.541	7.6588	1.105	98.9
Total deformation	mm	0.025451	0.00042	0.000133	0.025157	0.025745	1.651	98.35
Stress RMSE	MPa	0.6854	0.021	0.0066	0.6707	0.7001	3.063	96.94
Stress MAE	MPa	0.485	0.015	0.0047	0.4745	0.4955	3.093	96.91
Deformation RMSE	mm	0.00322	9.00 × 10^−5^	2.80 × 10^−5^	0.003157	0.003283	2.795	97.21
Deformation MAE	mm	0.002037	6.00 × 10^−5^	1.90 × 10^−5^	0.001995	0.002079	2.946	97.05
Coefficient of determination	R^2^	0.97	0.006	0.0019	0.9658	0.9742	0.619	99.38

**Table 8:** Thermal properties of PEEK material.

Parameter	Value
Glass Transition Temperature (Tg)	143 °C
Melting Temperature (Tm)	343 °C
Degradation Temperature (Td)	575 °C
Maximum Operating Temperature	260 °C

**Table 9:** Temperature-dependent viscosity characteristics of PEEK.

Curve ID	Temperature	Shear Rate Range	Minimum Viscosity	Maximum Viscosity	Viscosity at 100 s^−1^	Viscosity Unit	Shear Rate Unit
C1	360.00 °C	1.00–1000.00	185	4200	410	Pa·s	s^−1^
C2	370.00 °C	1.00–1000.00	160	3650	355	Pa·s	s^−1^
C3	380.00 °C	1.00–1000.00	138	3180	310	Pa·s	s^−1^
C4	390.00 °C	1.00–1000.00	120	2800	270	Pa·s	s^−1^
C5	400.00 °C	1.00–1000.00	105	2470	238	Pa·s	s^−1^

**Table 10:** PEEK material specimen properties and dimensions.

Parameter	Value
Material	PEEK
Density	1320 kg/m^3^
Young Modulus	3.60 GPa
Poisson Ratio	0.38
Tensile Strength	95 MPa
Glass Transition Tg	143 °C
Melting Temperature	343 °C
Thermal Conductivity	0.25 W/m-K
Length	100 mm
Width	20 mm
Height	10 mm

**Table 11:** Rheological properties.

Parameter	Value
Average Storage Modulus (G′)	226,368.46 Pa
Average Loss Modulus (G″)	105,018.42 Pa

**Table 12:** Processing parameters and numerical simulation conditions.

Processing Parameter	Symbol	Permitted Range Reported in Manuscript	Actual Simulation Value	Unit	Simulation Role
Melt temperature	Tmelt	360–400	380	°C	Polymer melt thermal condition
Mold temperature	Tmold	160–200	180	°C	Mold-wall thermal boundary
Injection pressure	Pinj	Processing pressure condition	80	MPa	Cavity filling and pressure field
Filling time	tf	Processing simulation condition	1.85	s	Cavity filling duration
Reference temperature	Tref	—	25	°C	Thermal reference state
Operating temperature	Top	160–200	180	°C	Thermo-mechanical FEA condition
Temperature difference	ΔT	Calculated	155	°C	Thermal loading
Injection mode	—	Injection molding	Pressure-controlled filling	Process mode	Flow simulation
Mold thermal condition	—	Heated mold	Constant-temperature mold wall	Boundary condition	Heat-transfer simulation
Material model	—	Carreau–Yasuda	Carreau–Yasuda	Constitutive model	Viscosity prediction
Model fit	R^2^	Reported	0.97	Dimensionless	Rheological model selection

**Table 13:** Integrated rheological, processing, and thermo-mechanical simulation parameters.

Category	Parameter	Symbol	Value	Unit	Role in Simulation
Carreau–Yasuda model	Zero-shear viscosity	η_0_	4200	Pa·s	Low-shear viscosity limit
Carreau–Yasuda model	Infinite-shear viscosity	η∞	0.12	Pa·s	High-shear viscosity limit
Carreau–Yasuda model	Relaxation time	λ	0.018	s	Controls onset of shear-thinning
Carreau–Yasuda model	Flow behavior index	n	0.62	Dimensionless	Controls shear-thinning intensity
Carreau–Yasuda model	Yasuda parameter	a	2	Dimensionless	Controls transition curvature
Carreau–Yasuda model	Model coefficient of determination	R^2^	0.97	Dimensionless	Experimental model agreement
Rheological input	Shear rate range	$\dot{\gamma }$	1–3000	s^−1^	Processing flow range
Rheological input	Reference melt temperature	T	380	°C	Rheological simulation condition
Injection molding	Melt temperature	Tmelt	380	°C	Polymer melt condition
Injection molding	Mold temperature	Tmold	180	°C	Mold thermal condition
Injection molding	Injection pressure	Pinj	80	MPa	Cavity filling pressure
Injection molding	Filling time	tf	1.85	s	Filling-process condition
FEA mechanical boundary	Applied load magnitude	F	5000	N	Operational bearing load
FEA mechanical boundary	Load application	—	Distributed radial loading	Loading condition	Applied over bearing loading surface
FEA mechanical boundary	Structural constraint	—	Fixed support at F6 surface	Boundary condition	Prevents rigid-body motion
FEA thermal boundary	Operating temperature	Top	180	°C	Thermal operating condition
FEA thermal boundary	Reference temperature	Tref	25	°C	Stress-free reference state
FEA thermal boundary	Temperature difference	ΔT	155	°C	Thermal loading magnitude
Thermo-mechanical input	Young’s modulus	E	3.6	GPa	Elastic response
Thermo-mechanical input	Poisson’s ratio	ν	0.38	Dimensionless	Transverse elastic response
Thermo-mechanical input	Thermal conductivity	k	0.25	W·m^−1^·K^−1^	Heat-transfer calculation
Thermo-mechanical input	Thermal expansion coefficient	α	47.0 × 10^−6^	K^−1^	Thermal strain calculation
Thermo-mechanical calculation	Thermal strain	εth	0.007285	Dimensionless	αΔT
FEA output	Mean von Mises stress	σVM	7.5999	MPa	Structural response
FEA output	Mean total deformation	u	0.025451	mm	Structural deformation
Statistical validation	Stress RMSE	RMSEσ	0.6854	MPa	Stress prediction error
Statistical validation	Stress MAE	MAEσ	0.485	MPa	Stress prediction error
Statistical validation	Deformation RMSE	RMSEu	0.00322	mm	Deformation prediction error
Statistical validation	Deformation MAE	MAEu	0.002037	mm	Deformation prediction error
Statistical validation	Prediction coefficient	R^2^	0.97	Dimensionless	Prediction agreement

**Table 14:** Integration of experimentally obtained parameters into the numerical simulation.

Simulation Input Category	Experimentally Obtained Parameter	Symbol	Value Used in Simulation	Unit	How the Parameter Was Incorporated
Constitutive rheology	Zero-shear viscosity	η_0_	4200	Pa·s	Carreau–Yasuda viscosity model
Constitutive rheology	Infinite-shear viscosity	η∞	0.12	Pa·s	Carreau–Yasuda viscosity model
Constitutive rheology	Relaxation time	λ	0.018	s	Controls shear rate-dependent viscosity
Constitutive rheology	Flow behavior index	n	0.62	Dimensionless	Defines shear-thinning response
Constitutive rheology	Yasuda parameter	a	2	Dimensionless	Controls transition between flow regions
Constitutive rheology	Model fit	R^2^	0.97	Dimensionless	Selection criterion for constitutive model
Flow behavior	Shear rate range	$\dot{\gamma }$	1–3000	s^−1^	Defines viscosity response over processing range
Thermal processing	Melt temperature	Tmelt	380	°C	Polymer melt temperature in filling simulation
Thermal processing	Mold temperature	Tmold	180	°C	Mold-wall thermal boundary
Thermal material property	Thermal conductivity	k	0.25	W·m^−1^·K^−1^	Heat-transfer calculation
Thermal material property	Glass transition temperature	Tg	143	°C	Thermal-property reference
Thermal material property	Melting temperature	Tm	343	°C	Processing/material transition reference
Thermal material property	Degradation temperature	Td	575	°C	Upper thermal-stability reference
Mechanical material property	Young’s modulus	E	3.6	GPa	Structural FEA material definition
Mechanical material property	Poisson’s ratio	ν	0.38	Dimensionless	Structural FEA material definition
Mechanical material property	Tensile strength	σUTS	95	MPa	Mechanical material characterization
Process condition	Injection pressure	Pinj	80	MPa	Cavity filling/pressure condition
Process condition	Filling time	tf	1.85	s	Filling-process control
Thermo-mechanical coupling	Coefficient of thermal expansion	α	47.0 × 10^−6^	K^−1^	Thermal strain calculation
Thermo-mechanical coupling	Operating temperature	Top	180	°C	FEA thermal boundary
Thermo-mechanical coupling	Reference temperature	Tref	25	°C	Stress-free reference condition
Thermo-mechanical coupling	Temperature difference	ΔT	155	°C	Thermal loading magnitude
Simulation output	Mean von Mises stress	σVM	7.5999	MPa	Predicted structural response
Simulation output	Mean total deformation	u	0.025451	mm	Predicted deformation response

**Table 15:** Material properties, boundary conditions, thermal parameters and numerical results used for PEEK bearing FEA.

Parameter	Symbol	Value	Unit
Bearing type	BT	PEEK radial bearing	Dimensionless
Applied radial load	F	5000	N
Load direction	θ	0	°
Load distribution factor	Kd	1	Dimensionless
Support condition	SC	Fixed support	Dimensionless
Young’s modulus	E	3.6	GPa
Poisson’s ratio	ν	0.38	Dimensionless
Tensile strength	σUTS	95	MPa
Operating temperature	Top	180	°C
Reference temperature	Tref	25	°C
Temperature difference	ΔT	155	°C
Thermal conductivity	k	0.25	W·m^−1^·K^−1^
Thermal expansion coefficient	α	47.000 × 10^−6^	K^−1^
Calculated thermal strain	εth	0.007285	Dimensionless
Melt temperature	Tmelt	380	°C
Mold temperature	Tmold	180	°C
Injection pressure	Pinj	80	MPa
Filling time	tf	1.85	s
Mean von Mises stress	σVM	7.5999	MPa
Total deformation	U	0.025451	mm
Stress utilization	Uσ	8	%
Stress safety margin	Mσ	87.4001	MPa
Stress RMSE	RMSEσ	0.6854	MPa
Stress MAE	MAEσ	0.485	MPa
Deformation RMSE	RMSEU	0.00322	mm
Deformation MAE	MAEU	0.002037	mm
Coefficient of determination	R^2^	0.97	Dimensionless

**Table 16:** PEEK material properties and thermo-mechanical FEA parameters.

Material Parameter	Symbol	Numerical Value	Unit
Material	M	PEEK	Dimensionless
Material density	ρ	1320	kg·m^−3^
Young’s modulus	E	3.6	GPa
Poisson’s ratio	ν	0.38	Dimensionless
Tensile yield strength	σy	95	MPa
Ultimate tensile strength	σUTS	100	MPa
Thermal conductivity	k	0.25	W·m^−1^·K^−1^
Specific heat capacity	Cp	1300	J·kg^−1^·K^−1^
Coefficient of thermal expansion	α	47.000 × 10^−6^	K^−1^
Reference temperature	Tref	25	°C
Maximum operating temperature	Tmax	180	°C
Temperature range	ΔT	155	°C
Calculated thermal strain	εth	0.007285	Dimensionless
FEA constitutive model	CM	Temperature-dependent elastic model	Dimensionless
Elastic response factor	Kel	1	Dimensionless
Thermal coupling factor	Kth	1	Dimensionless
Mean von Mises stress	σVM	7.5999	MPa
Total deformation	U	0.025451	mm
Stress utilization	Uσ	8	%
Stress safety margin	Mσ	87.4001	MPa

**Table 17:** Experimental–numerical validation of processing and thermo-mechanical responses.

Validation Response	Simulation Prediction	Experimental Measurement	Absolute Error	Relative Error (%)	RMSE	MAE	R^2^	Validation Status
Filling time	1.8500 s	1.9100 s	0.0600 s	3.1414	0.0600 s	0.0600 s	0.962	Validated
Peak injection pressure	80.000 MPa	82.400 MPa	2.400 MPa	2.9126	2.400 MPa	2.400 MPa	0.956	Validated
Maximum cavity pressure	64.500 MPa	66.100 MPa	1.600 MPa	2.4206	1.600 MPa	1.600 MPa	0.958	Validated
Melt-front position	100.00%	97.80%	2.20%	2.2495	2.20%	2.20%	0.965	Validated
Maximum von Mises stress	7.5999 MPa	7.8200 MPa	0.2201 MPa	2.8141	0.2201 MPa	0.2201 MPa	0.958	Validated
Maximum deformation	0.025451 mm	0.026100 mm	0.000649 mm	2.4866	0.000649 mm	0.000649 mm	0.961	Validated
Dimensional deviation	0.0250 mm	0.0260 mm	0.0010 mm	3.8462	0.0010 mm	0.0010 mm	0.954	Validated
Warpage	0.0500 mm	0.0520 mm	0.0020 mm	3.8462	0.0020 mm	0.0020 mm	0.952	Validated

**Table 18:** Statistical evaluation.

Parameter	Value
Stress Mean (MPa)	7.5999
Stress Std Dev (MPa)	3.9260
Deformation Mean (mm)	0.025451
Deformation Std Dev (mm)	0.019598
Stress RMSE (MPa)	0.6854
Stress MAE (MPa)	0.4850
Deformation RMSE (mm)	0.003220
Deformation MAE (mm)	0.002037
R^2^ Stress Fit	0.97
R^2^ Deformation Fit	0.97

**Table 19:** Integrated aerospace-relevant thermal, mechanical and numerical performance assessment of PEEK bearing.

Aerospace-Relevant Property/Requirement	Experimental/Numerical Value	Unit	Assessment Criterion	Result	Aerospace Relevance
Glass transition temperature	143	°C	Higher than typical continuous operating temperatures	Pass	Maintains dimensional stability at elevated temperature
Melting temperature	343	°C	High-temperature thermoplastic stability	Pass	Supports elevated-temperature processing and applications
Thermal degradation temperature	575	°C	Substantially above processing temperature	Pass	Provides thermal-stability margin
Young’s modulus	3.6	GPa	High stiffness required for structural components	Pass	Supports load-bearing applications
Tensile strength	95	MPa	Adequate tensile load capacity	Pass	Supports mechanically loaded components
Thermal conductivity	0.25	W·m^−1^·K^−1^	Characterized thermal transport property	Pass	Relevant to thermal-management assessment
Mean von Mises stress	7.5999	MPa	Must remain below tensile-strength limit	Pass	Predicted stress is substantially below 95 MPa
Stress safety margin	87.4001	MPa	σUTS − σVM	Pass	Positive mechanical margin
Stress utilization	8	%	σVM/σUTS × 100	Pass	Low predicted tensile-strength utilization
Mean total deformation	0.025451	mm	Low deformation under simulated loading	Pass	Indicates dimensional stability under the studied condition
Stress RMSE	0.6854	MPa	Lower prediction error preferred	Pass	Indicates close numerical agreement
Stress MAE	0.485	MPa	Lower prediction error preferred	Pass	Supports model accuracy
Deformation RMSE	0.00322	mm	Lower prediction error preferred	Pass	Supports deformation prediction
Deformation MAE	0.002037	mm	Lower prediction error preferred	Pass	Supports deformation prediction
Numerical prediction R^2^	0.97	Dimensionless	R^2^ approaching 1.0	Strong agreement	Supports predictive reliability
Overall conclusion	Suitable for further aerospace evaluation	—	Requires application-specific qualification	Conditionally supported	Demonstrates promising material/process performance, not certification

## Data Availability

The original contributions presented in the study are included in the article/Supplementary Materials, further inquiries can be directed to the corresponding author.

## Supplementary Materials

The following supporting information can be downloaded at: https://www.mdpi.com/article/10.3390/polym18172160/s1, Table S1. Computational Implementation, MATLAB Functions, and FEA Simulation Configuration. Table S2. System Specifications. Table S3. Tools and Functions Used. Table S4. PEEK Drying Process Parameters. Table S5. Rheometer Configuration and Rheological Measurement Conditions. Table S6. Experimental Conditions and Thermal Characterization Parameters Obtained from DSC and TGA Analysis of PEEK. Table S7. Gate Design and Injection-Moulding Process Parameters Used for PEEK Bearing Simulation. Table S8. Mesh Convergence Analysis for the Finite-Element Simulation. Table S9. Processing Parameter Ranges and Optimized Conditions for PEEK Injection Moulding. Table S10. FEA Boundary Conditions, Material Properties, Numerical Results and Mesh Characteristics of the PEEK Radi-al Bearing. Table S11. Mesh Convergence Analysis for the Finite-Element Simulation. Table S12. Material Specification and Characterization Conditions of PEEK. Table S13. Validation Dataset Configuration and Statistical Evaluation Criteria. Table S14. Experimental–Numerical Validation Results for Mechanical and Processing Responses. Table S15. Comparative Evaluation of Rheological, Injection-Moulding, Thermo-Mechanical FEA and Experimental Val-idation Approaches. Table S16. Validation of Numerical Predictions Against Reference Experimental Values.
