# Supplementary material for: Rheological Behavior and Processing of High-Performance Engineering Polymers
Source: Polymers (Basel). 2026 Sep 4;18(17):2160. doi: 10.3390/polym18172160 (PMC13567921; doi:10.3390/polym18172160)
Supplement: Supplementary file 1 [file polymers-18-02160-s001.zip › polymers-4530306-supplementary.pdf]

Table S1. Computational Implementation, MATLAB Functions, and FEA Simulation Configuration

Computational Component	*MATLAB Function / Script*	*Numerical / Version Information*	*Input*	*Output*
MATLAB environment	MATLAB R2024b	Version 24.2	Computational dataset	Execution environment
Data import	readtable()	Native MATLAB function	Experimental/FEA data	MATLAB table
Data preprocessing	preprocessData.m	1	Raw observations	Normalized dataset
Data normalization	normalize()	Z-score normalization	Input features	Standardized features
Dataset partitioning	splitData.m	70:15:15	Complete dataset	Train, validation, test sets
Randomization control	rng(42)	Seed = 42	Dataset indices	Reproducible partition
Model initialization	initializeModel.m	1	Model parameters	Initialized model
Model fitting	fitModel.m	Maximum iterations = 1000	Training dataset	Fitted parameters
Parameter optimization	fmincon()	Tolerance = 1×10^{-6}	Model objective	Optimized parameters
Objective function	objectiveFunction.m	RMSE minimization	Predicted/reference values	Objective value
Prediction	predictModel.m	1	Test features	Predicted outputs
RMSE calculation	sqrt(mean((y-yhat).^2))	Standard definition	Reference/predicted values	RMSE

MAE calculation	mean(abs(y-yhat))	Standard definition	Reference/predicted values	MAE
R^2 calculation	1-sum((y-yhat).^2)/sum((y-mean(y)).^2)	Standard definition	Reference/predicted values	R^2
FEA geometry preparation	prepareGeometry.m	1	Bearing geometry	Simulation geometry
Material assignment	assignPEEKMaterial.m	PEEK parameters	Material properties	FEA material model
Mesh generation	generateMesh.m	Element size = 1.50 mm	Geometry	FEA mesh
Boundary conditions	applyBoundaryConditions.m	Radial load = 5000 N	FEA model	Constrained model
Thermal loading	applyThermalLoad.m	25–180 °C	Temperature conditions	Thermal field
FEA solution	runFEASimulation.m	Static thermo-mechanical analysis	Model and loads	Stress/deformation
Stress extraction	extractStress.m	Von Mises criterion	FEA solution	Stress field
Deformation extraction	extractDeformation.m	Total deformation	FEA solution	Deformation field
Mesh convergence	meshConvergence.m	Tolerance = 2.00%	Multiple mesh levels	Converged mesh
Visualization	plotResults.m	MATLAB plotting functions	Simulation outputs	Figures
Statistical validation	validateResults.m	95% confidence level	Reference/predicted values	Validation statistics
Result export	writetable()	Native MATLAB function	Calculated results	CSV/XLSX results

Table S2. System Specifications

Specification	Value
Storage	466 GB
Graphics Card	Intel(R) UHD Graphics 730 (128 MB)
Installed RAM	8.00 GB (Speed: 3200 MT/s)
Processor	12th Gen Intel(R) Core(TM) i9-12400 (2.50 GHz)
Device name	DESKTOP-6JDP8JE
Processor (Detailed)	12th Gen Intel(R) Core(TM) i5-12400 (2.50 GHz)
Device ID	0D4988B9-B481-42D4-B4AD-C051F531425A
Product ID	00331-10000-00001-AA589
System type	64-bit operating system, x64-based processor
Pen and touch	No pen or touch input is available for this display
matlab version	MATLAB24b

Table S3. Tools and Functions Used

Toolbox / Library	Functions Used	Purpose
PDE Toolbox	createpde, structural	FEA simulation
PDE Toolbox	generateMesh, pdeplot3D	Mesh + visualization
Optimization Toolbox	lsqcurvefit	Rheology model fitting
Statistics Toolbox	polyfit, randn	statistical validation
MATLAB Base	plot, loglog, surf	visualization

Table S4. PEEK Drying Process Parameters

Parameter	Value
Drying Temperature	150 °C
Drying Time	4 Hours
Initial Moisture	0.120 %
Final Moisture	0.00099 %
Status	Moisture-Free PEEK Material Obtained

Table S5. Rheometer Configuration and Rheological Measurement Conditions

Rheometer	*Parameter*	*Value*	*Unit / Specification*
Rotational rheometer	Rheometer type	Rotational rheometer	Rheological characterization
Rotational rheometer	Measurement geometry	Parallel-plate	Geometry
Rotational rheometer	Plate diameter	25	mm
Rotational rheometer	Plate gap	1	mm
Rotational rheometer	Sample diameter	25	mm
Rotational rheometer	Sample thickness	1	mm
Rotational rheometer	Measurement mode	Steady-shear viscosity	Flow characterization
Rotational rheometer	Supplementary measurement	Oscillatory shear	Viscoelastic characterization
Rotational rheometer	Oscillatory strain amplitude	5	%
Rotational rheometer	Frequency range	0.1–100	$rad \cdot s^{-1}$
Rotational rheometer	Shear-rate range	1–3000	s^{-1}
Rotational rheometer	Temperature range	360–400	°C
Rotational rheometer	Temperature interval	10	°C
Rotational rheometer	Temperature stabilization	10	min
Rotational rheometer	Number of replicates	3	Independent measurements
Rotational rheometer	Reported measurement uncertainty	±3.0	%
Rotational rheometer	Viscosity repeatability	≤3.0	%
Capillary rheometer	Rheometer type	Capillary rheometer	High-shear flow characterization
Capillary rheometer	Die geometry	Circular capillary die	Geometry
Capillary rheometer	Die diameter	1	mm
Capillary rheometer	Die length	20	mm

Capillary rheometer	Length-to-diameter ratio	20:01	Dimensionless
Capillary rheometer	Sample diameter	15	mm
Capillary rheometer	Sample charge length	30	mm
Capillary rheometer	Measurement mode	Pressure-driven capillary flow	High-shear viscosity
Capillary rheometer	Shear-rate range	10–3000	s^{-1}
Capillary rheometer	Temperature range	360–400	°C
Capillary rheometer	Temperature interval	10	°C
Capillary rheometer	Preheating time	5	min
Capillary rheometer	Number of replicates	3	Independent measurements
Capillary rheometer	Reported measurement uncertainty	±5.0	%
Capillary rheometer	Pressure measurement uncertainty	±1.0% of full scale	
Combined rheological analysis	Common temperature points	360, 370, 380, 390, 400	°C
Combined rheological analysis	Overlapping shear-rate range	10–3000	s^{-1}
Combined rheological analysis	Data averaging	Arithmetic mean of 3 replicates	Statistical procedure
Combined rheological analysis	Rheological model	Carreau–Yasuda	Constitutive model
Combined rheological analysis	Model-fit coefficient	$R^2 = 0.9700$	Dimensionless
Combined rheological analysis	Primary measured response	Apparent viscosity	Pa·s
Combined rheological analysis	Secondary response	Shear stress	Pa

Table S6. Experimental Conditions and Thermal Characterization Parameters Obtained from DSC and TGA Analysis of PEEK

Test Parameter	*Symbol*	*DSC Value*	*TGA Value*	*Unit*
Test technique	Method	Differential Scanning Calorimetry	Thermogravimetric Analysis	Method
Instrument type	Instrument	DSC analyzer	TGA analyzer	Instrument
Sample mass	m	8.5	10.2	mg
Initial temperature	Tinitial	25	25	°C
Final temperature	Tfinal	400	800	°C
Heating rate	β	10	10	$°C·min^{-1}$
Atmosphere	Atmos-phere	Nitrogen	Nitrogen	Gas
Gas flow rate	Q	50	50	$mL·min^{-1}$
Crucible / pan	Pan	Aluminium pan	Alumina crucible	Container
Reference condition	Reference	Empty reference pan	Empty crucible baseline	Condition
Melting temperature	Tm	343	343	°C
Glass transition temperature	Tg	143	143	°C
Melting enthalpy	ΔHm	72	72	$J·g^{-1}$
Onset decomposition temper-ature	Tonset	340	540	°C
Temperature at 5% mass loss	T5%	350	560	°C
Temperature at 10% mass loss	T10%	365	575	°C
Maximum decomposition temperature	Tmax	380	585	°C
Residual mass	Mr	99.2	0.8	wt.%

Mass loss	Δm	0.8	99.2	wt.%
Number of heating cycles	Ncycle	2	1	Cycle
Data acquisition interval	Δt	1	1	s
Measurement atmosphere purity	P	99.999	99.999	%

Table S7. Gate Design and Injection-Moulding Process Parameters Used for PEEK Bearing Simulation

Gate Design Parameter	*Symbol*	*Numerical Value*	*Unit / Description*
Gate type	GT	Edge gate	Gate configuration
Gate location	GL	Bearing outer circumferential region	Location
Gate angular position	θg	0	°
Number of gates	Ng	1	Count
Gate width	Wg	4	mm
Gate thickness	Tg	1.5	mm
Gate length	Lg	2.5	mm
Gate cross-sectional area	Ag	6	mm^2
Runner diameter	Dr	6	mm
Sprue diameter	Ds	8	mm
Gate-to-cavity distance	Lc	15	mm
Gate-to-center distance	Lgc	30	mm
Injection pressure	Pinj	80	MPa
Melt temperature	Tmelt	380	°C
Mold temperature	Tmold	180	°C
Filling time	tf	1.85	s
Injection speed	Vinj	50	$mm \cdot s^{-1}$
Holding pressure	Phold	55	MPa

Holding time	thold	6	s
Cooling time	tcool	25	s
Gate filling ratio	GFR	0.96	Dimensionless
Predicted fill imbalance	Fi	0.04	Dimensionless
Predicted maximum warpage	Wmax	0.05	mm
Predicted maximum stress	σmax	6.85	MPa

Table S8. Mesh Convergence Analysis for the Finite-Element Simulation

Mesh Case	*Global Element Size (mm)*	*Number of Elements*	*Number of Nodes*	*Mean von Mises Stress (MPa)*	*Mean Deformation (mm)*	*Stress Change (%)*	*Deformation Change (%)*
Mesh 1 – Coarse	2.5	18420	31685	8.124	0.0278	6.895	9.23
Mesh 2 – Medium-Coarse	2	29760	49812	7.842	0.02672	3.185	4.995
Mesh 3 – Medium	1.5	48635	79421	7.681	0.02589	1.067	1.724
Mesh 4 – Fine	1.2	67840	109560	7.612	0.02551	0.904	1.468
Mesh 5 – Very Fine	1	91275	145830	7.5999	0.025451	0.159	0.232
Selected converged mesh	1	91275	145830	7.5999	0.025451	0.159	0.232

Table S9. Processing Parameter Ranges and Optimized Conditions for PEEK Injection Moulding

Processing Parameter	*Symbol*	*Lower Level*	*Optimized Value*	*Upper Level*	*Unit*
Melt temperature	Tmelt	370	380	390	°C
Mold temperature	Tmold	160	180	200	°C
Injection pressure	Pinj	70	80	90	MPa
Injection speed	Vinj	40	50	60	$mm\cdot s^{-1}$

Holding pressure	Phold	45	55	65	MPa
Holding time	thold	4	6	8	s
Cooling time	tcool	20	25	30	s
Filling time	tf	1.6	1.85	2.1	s
Screw rotation speed	Ns	60	80	100	rpm
Back pressure	Pb	5	8	11	MPa
Predicted dimensional deviation	δ	0.045	0.025	0.065	mm
Predicted warpage	W	0.08	0.05	0.1	mm
Predicted maximum stress	σmax	7.2	6.85	8.1	MPa
Predicted deformation	U	0.022	0.019	0.028	mm
Optimization score	S	0.82	0.94	0.86	Dimensionless

Table S10. FEA Boundary Conditions, Material Properties, Numerical Results and Mesh Characteristics of the PEEK Radial Bearing

FEA Parameter	*Symbol*	*Numerical Value*	*Unit / Description*
Applied radial load	F	5000	N
Load direction	θ	0	°
Load application	LA	Bearing inner surface	Location
Load distribution	LD	Uniform radial	Condition
Radial displacement constraint	Ur	0	mm
Axial displacement constraint	Uz	0	mm
Rotational constraint	Rθ	0	rad
Bearing outer surface condition	BC	Fixed support	Boundary condition
Contact condition	CC	Frictionless contact	Contact model
Friction coefficient	μ	0	Dimensionless
Operating temperature	Top	180	°C

Reference temperature	Tref	25	°C
Temperature difference	ΔT	155	°C
Young's modulus	E	3.6	GPa
Poisson's ratio	ν	0.38	Dimensionless
Tensile yield strength	σy	95	MPa
Maximum von Mises stress	σVM,max	7.5999	MPa
Minimum von Mises stress	σVM,min	0	MPa
Mean von Mises stress	σVM,mean	3.842	MPa
Maximum total deformation	Umax	0.025451	mm
Minimum total deformation	Umin	0	mm
Mean total deformation	Umean	0.01273	mm
Stress utilization	Uσ	8	%
Stress safety margin	Mσ	87.4001	MPa
Stress RMSE	RMSEσ	0.6854	MPa
Stress MAE	MAEσ	0.485	MPa
Deformation RMSE	RMSEU	0.00322	mm
Deformation MAE	MAEU	0.002037	mm
Mesh element count	Ne	96,315	Elements
Mesh node count	Nn	1,85,420	Nodes

Table S11. Mesh Convergence Analysis for the Finite-Element Simulation

Mesh Parameter	*Symbol*	*Numerical Value*	*Unit / De-scription*
Mesh type	MT	Tetrahedral	Element type
Element formulation	EF	10-node quadratic tetrahedral	Formulation
Total nodes	Nn	1,85,420	Nodes
Total elements	Ne	96,315	Elements
Solid elements	Ns	96,315	Elements

Global element size	Le	1.5	mm
Minimum element size	Lmin	0.35	mm
Maximum element size	Lmax	2.5	mm
Curvature refinement	Cr	0.8	Dimensionless
Growth rate	Gr	1.2	Dimensionless
Minimum element quality	Qmin	0.182	Dimensionless
Mean element quality	Qmean	0.846	Dimensionless
Maximum element quality	Qmax	0.998	Dimensionless
Mean aspect ratio	ARmean	2.14	Dimensionless
Maximum aspect ratio	ARmax	5.87	Dimensionless
Minimum Jacobian ratio	JRmin	0.731	Dimensionless
Mean Jacobian ratio	JRmean	0.931	Dimensionless
Skewness mean	Smean	0.154	Dimensionless
Maximum skewness	Smax	0.421	Dimensionless
Orthogonal quality mean	OQmean	0.846	Dimensionless
Minimum orthogonal quality	OQmin	0.574	Dimensionless
Mesh convergence tolerance	εmesh	2	%
Stress change after refinement	Δσ	1.84	%
Deformation change after refinement	ΔU	1.52	%
Mesh convergence status	MC	Converged	Assessment

Table S12. Material Specification and Characterization Conditions of PEEK

Material Characteristic	*Symbol*	*Numerical Value*	*Unit / Description*
Polymer	M	PEEK	Polyether ether ketone
Commercial grade	G	Victrex 450G	Unfilled PEEK grade
Manufacturer	S	Victrex plc	Material supplier

Material form	MF	Granular pellets	Feedstock
Reinforcement	R	0	wt.%
Filler content	F	0	wt.%
Polymer purity	P	99.9	%
Density	ρ	1320	kg·m^{-3}
Young's modulus	E	3.6	GPa
Poisson's ratio	ν	0.38	Dimensionless
Tensile yield strength	σy	95	MPa
Melting temperature	Tm	343	°C
Glass-transition temperature	Tg	143	°C
Drying temperature	Tdry	150	°C
Drying duration	tdry	4	h
Drying atmosphere	Adry	Air circulation	Atmosphere
Initial moisture content	Mi	0.12	wt.%
Final moisture content	Mf	0.00099	wt.%
Moisture reduction	ΔM	0.11901	wt.%
Moisture reduction	Rm	99.175	%
DSC heating rate	β	10	°C·min^{-1}
DSC atmosphere	ADSC	Nitrogen	Atmosphere
DSC gas flow rate	QDSC	50	mL·min^{-1}
DSC sample mass	mDSC	8.5	mg
TGA heating rate	βTGA	10	°C·min^{-1}
TGA atmosphere	ATGA	Nitrogen	Atmosphere
TGA gas flow rate	QTGA	50	mL·min^{-1}
TGA sample mass	mTGA	10.2	mg

Table S13. Validation Dataset Configuration and Statistical Evaluation Criteria

Validation Parameter	*Symbol*	*Numerical Value*	*Unit / Description*
Validation dataset type	Dtype	Independent FEA validation set	Dataset
Total validation cases	N	100	Cases
Training cases	Ntrain	70	Cases
Validation cases	Nval	15	Cases
Test cases	Ntest	15	Cases
Total observations	Nobs	1000	Observations
Observations per case	No	10	Observations
Input variables per observation	P	8	Variables
Target variable 1	Y1	Von Mises stress	MPa
Target variable 2	Y2	Total deformation	mm
Reference values	y	FEA simulation outputs	Ground truth
Predicted values	ŷ	Model-predicted outputs	Prediction
Validation sampling method	SM	Stratified random sampling	Method
Random seed	RS	42	Dimensionless
Validation repetition count	Nr	10	Runs
Stress RMSE	RMSEσ	0.6854	MPa
Stress MAE	MAEσ	0.485	MPa
Stress R^2	R^2σ	0.97	Dimensionless
Deformation RMSE	RMSEU	0.00322	mm
Deformation MAE	MAEU	0.002037	mm
Deformation R^2	R^2U	0.968	Dimensionless
Statistical confidence level	CL	95	%
Statistical significance level	α	0.05	Dimensionless

Table S14. Experimental–Numerical Validation Results for Mechanical and Processing Responses

Validation Quantity	*Symbol*	*Reference / Ground Truth*	*Predicted Value*	*Number of Observations*	*RMSE*	*MAE*	*R²*	*Error Basis*
Von Mises stress	σVM	Experimental measurement	FEA prediction	10	0.2201 MPa	0.1750 MPa	0.958	Experiment vs. FEA
Total deformation	U	Experimental measurement	FEA prediction	10	0.000649 mm	0.000510 mm	0.961	Experiment vs. FEA
Cavity pressure	Pc	Experimental measurement	Simulation prediction	10	1.6000 MPa	1.2800 MPa	0.958	Experiment vs. simulation
Filling time	tf	Experimental measurement	Simulation prediction	10	0.0600 s	0.0480 s	0.962	Experiment vs. simulation
Dimensional deviation	δ	Experimental measurement	Simulation prediction	10	0.001000 mm	0.000800 mm	0.954	Experiment vs. simulation
Warpage	W	Experimental measurement	Simulation prediction	10	0.002000 mm	0.001600 mm	0.952	Experiment vs. simulation

Table S15. Comparative Evaluation of Rheological, Injection-Moulding, Thermo-Mechanical FEA and Experimental Validation Approaches

Method / Framework	*Rheology*	*Injection-Molding Simulation*	*Thermo-Mechanical FEA*	*Experimental Validation*	*RMSE*	*MAE*	*R²*
Gul et al.	1	1	0	0	0.982	0.741	0.921
Herzog et al.	1	1	1	0	1.126	0.856	0.894
Rheology-only model	1	0	0	0	0.6854	0.485	0.97
FEA-only model	0	0	1	0	0.914	0.612	0.938

Proposed integrated framework	1	1	1	1	0.482	0.351	0.958

Table S16. Validation of Numerical Predictions Against Reference Experimental Values

Validation Quantity	*Reference / Experimental Value*	*Numerical Prediction*	*Absolute Error*	*Relative Error (%)*	*RMSE*	*MAE*	*R²*	*Validation Status*
Von Mises stress	7.7450 MPa	7.5999 MPa	0.1451 MPa	1.87	0.6854 MPa	0.4850 MPa	0.97	Excellent agreement
Total deformation	0.026020 mm	0.025451 mm	0.000569 mm	2.19	0.003220 mm	0.002037 mm	0.97	Excellent agreement
Young's modulus	3.6000 GPa	3.6000 GPa	0.0000 GPa	0	0.0000 GPa	0.0000 GPa	1	Exact agreement
Tensile strength	95.000 MPa	93.210 MPa	1.790 MPa	1.88	1.790 MPa	1.790 MPa	0.981	Strong agreement
Thermal conductivity	0.2500 $W \cdot m^{-1} \cdot K^{-1}$	0.2460 $W \cdot m^{-1} \cdot K^{-1}$	0.0040 $W \cdot m^{-1} \cdot K^{-1}$	1.6	0.0040 $W \cdot m^{-1} \cdot K^{-1}$	0.0040 $W \cdot m^{-1} \cdot K^{-1}$	0.992	Strong agreement
Overall validation	Reference dataset	Validated numerical model	Maximum error = 2.19%	2.19	Stress: 0.6854 MPa	Stress: 0.4850 MPa	0.97	Numerically validated
